# Resilience and its mediating role in the association between family functioning and symptom experience in kidney transplant recipients: A cross-sectional study

**DOI:** 10.1371/journal.pone.0361968

**Published:** 2026-11-12

**Authors:** Yan Tang, Jiefu Zhu, Jinke Li, Jiangqiao Zhou, Rong Wang, Tao Qiu

**Affiliations:** 1 Hemodialysis Center, The Sixth Hospital of Wuhan, Affiliated Hospital of Jianghan University, Wuhan, Hubei, China; 2 The Department of Organ Transplantation, Renmin Hospital of Wuhan University, Wuhan, Hubei, China; 3 The Department of Urology, Renmin Hospital of Wuhan University, Wuhan, Hubei, China; 4 Nursing Department, Renmin Hospital of Wuhan University, Wuhan, Hubei, China; Phramongkutklao College of Medicine, THAILAND

## Abstract

**Objective:**

To investigate the levels of family functioning, resilience, and symptom experience in kidney transplant recipients, and to examine the mediating role of resilience in the relationship between family functioning and symptom experience.

**Methods:**

A cross-sectional study was conducted from January to February 2025. A convenience sample of 333 kidney transplant recipients was recruited from a tertiary hospital in Wuhan, China. Participants completed the Chinese Version of the Resilience Scale for Kidney Transplantation, the Organ Transplant Symptom and Well-being Inventory, and the Family APGAR Index. Data were analyzed using correlation, hierarchical regression, and bootstrap mediation tests.

**Results:**

Participants reported a moderate level of symptom burden (OTSWI total score: 46.56 ± 23.86). Both resilience (*r* = –0.222, *p* < .01) and family functioning (*r* = –0.202, *p* < .01) were significantly correlated with lower symptom burden. After controlling for demographic and clinical variables, resilience (*β = −0.152, p = .005*) and family functioning (*β = −0.124, p = .022*) remained independent predictors. Mediation analysis showed that resilience was consistent with a partial mediating role in the relationship between family functioning and symptom experience, accounting for 18.4% of the total effect (indirect effect = –0.326, 95% *BCa CI* [–0.645, –0.081]).

**Conclusion:**

The findings are consistent with resilience partially mediating the association between family functioning and symptom experience in kidney transplant recipients. Assessing both family support and psychological resilience may help identify high-risk patients and inform integrated interventions aimed at alleviating symptom burden.Given the cross-sectional design, causal inference cannot be established.

## Introduction

Kidney transplantation is the treatment of choice for end-stage renal disease, offering patients a significant survival benefit and improved quality of life compared to long-term dialysis. However, even after a successful surgery, recipients face a multitude of long-term physiological and psychosocial challenges that extend beyond graft survival [1]. A significant burden stems from the experience of a complex array of symptoms, including but not limited to fatigue, gastrointestinal discomfort, sleep disturbances, anxiety, and depression [2,3]. This multifaceted symptom experience profoundly impacts patients’ physical well-being, daily functioning, and can exacerbate psychological distress, ultimately undermining the overall success of transplantation [3,4].

Family functioning, as a crucial component of social support, plays a pivotal role in influencing patient outcomes. Effective family functioning, characterized by cohesive communication, emotional support, and adaptive problem-solving, can provide tangible assistance and psychological comfort, thereby helping to mitigate the symptom burden for kidney transplant recipients [5,6]. While previous research has established a direct link between family functioning and quality of life [7], it is observed that some patients with apparently good family support still report poor outcomes [8]. This paradox suggests the existence of unexplored mediating mechanisms through which family support translates into individual health outcomes.

Psychological resilience may be a key mediator in this pathway. Defined as the ability to maintain psychological adaptation and positive adjustment in the face of adversity, trauma, or significant stress [9], resilience is increasingly recognized as a critical personal resource in chronic illness management. In kidney transplant populations, higher resilience is associated with improved medication adherence, reduced psychological distress, and enhanced quality of life [6,10]. However, most existing studies have focused on the independent effects of either family support or resilience. The specific pathway through which they interact to influence patients’ symptom experience remains unclear and warrants further investigation.

This study was guided by the Theory of Unpleasant Symptoms (TOUS) [11], which posits that symptom experience is multifactorial, arising from the confluence of physiological, psychological, and situational influences. We explicitly mapped our variables onto this framework: family functioning was treated as a situational factor, resilience as a psychological factor, and symptom experience as the core outcome. This mapping provided a theoretical basis for our central hypothesis—that the effect of the situational factor (family functioning) on symptoms is not only direct but also operates indirectly through the psychological factor (resilience). Thereby, the TOUS directly informed our analytical approach, specifically supporting the test of the mediation model.

### Aims and hypotheses

Therefore, to address the gap concerning how family functioning and resilience interact to influence symptom experience, this study aimed to investigate the mediating role of resilience in this relationship among kidney transplant recipients, guided by the TOUS. We hypothesized that: (1) better family functioning would be directly associated with less severe symptom experience; (2) greater resilience would be directly associated with less severe symptom experience; and (3) resilience would significantly mediate the relationship between family functioning and symptom experience.

## Materials and methods

### Study design

A cross-sectional, questionnaire-based study was conducted and reported following the Strengthening the Reporting of Observational Studies in Epidemiology (STROBE) guidelines. The STROBE checklist for cross-sectional studies is provided as S1 Checklist.

### Participants and settings

A convenience sample of kidney transplant recipients was recruited between January and February 2025 from the outpatient follow-up clinic of a tertiary Grade A hospital in Wuhan, China.

Inclusion criteria were: (1) aged 18 years or older; (2) having undergone kidney transplantation at least 3 months prior (a minimum of 3 months post-transplant was chosen to allow for recovery from the acute surgical phase and stabilization of immunosuppressive therapy); (3) medically stable, conscious, and able to communicate effectively. Exclusion criteria were: (1) recipients of multi-organ transplants; (2) a history of cognitive impairment or psychiatric disorders; (3) experiencing major comorbidities (defined as active malignancy, end-stage cardiac or pulmonary disease, or recent [within 6 months] myocardial infarction or stroke) or stressful life events (defined as death of a family member, divorce, job loss, or major financial crisis in the past 3 months). The transplant program at Renmin Hospital of Wuhan University follows a standard immunosuppression protocol (tacrolimus, mycophenolate mofetil, and corticosteroids). Routine follow-up visits occur at 1, 3, 6, 12 months post-transplant and annually thereafter. Formal psychosocial services are available on referral.

The sample size was estimated based on the requirement for multiple regression analysis, which recommends 5–10 participants per independent variable [12]. Our final hierarchical regression model contained 7 predictor variables (gender, 2 dummy variables for employment status, 3 dummy variables for monthly income, residence, complications, resilience, and family functioning). Based on the 5–10 events per variable rule, the required minimum sample size would be 35–70. Accounting for a potential 20% invalid response rate, we aimed to recruit at least 85 participants. A total of 335 questionnaires were distributed. After excluding two invalid responses (one failed attention check, one straight-line response pattern), 333 participants were included in the final analysis. This sample size is more than adequate for both regression and mediation analyses, and provides sufficient power to detect indirect effects with bootstrap methods.

### Ethical approval

This study was approved by the Medical Ethics Committee of the participating hospital (Approval No: WDRY2024-K282). Electronic informed consent was obtained from all participants prior to data collection.

### Data collection

Data were collected via an electronic questionnaire on the WJX.cn platform. Eligible participants were approached in the outpatient clinic. After a full explanation of the study, electronic informed consent was obtained. The questionnaire incorporated forced-response settings and an attention-check item to ensure data quality. Research team members monitored submission progress. Out of 335 questionnaires submitted, two were excluded (one due to a failed attention check and another for a straight-line response pattern), resulting in 333 valid responses (99.4% valid response rate).

### Measures

#### General information questionnaire.

A self-developed form collected socio-demographic and clinical data, including age, gender, education, employment status, monthly income, transplant duration, and complications. Complications were defined as any post-transplant medical event requiring hospitalization or significant treatment adjustment within the past 12 months, including but not limited to acute rejection, infection, cardiovascular events, or new-onset diabetes.

#### Resilience.

Psychological resilience was measured using the Chinese Version of the Resilience Scale for Kidney Transplantation (RS-KTPL-CV). This scale was cross-culturally adapted and validated by our research team in a prior study [13]. It comprises 22 items across four dimensions: positive attitude, medical management, social support, and health habits. Items are rated on a 4-point Likert scale from 1 (strongly disagree) to 4 (strongly agree), with total scores ranging from 22 to 88. Higher scores indicate greater resilience. In this study, the scale demonstrated excellent internal consistency (Cronbach’s α = .945).

#### Symptom experience.

The Chinese version of the Organ Transplant Symptom and Well-being Inventory (OTSWI) was used [14]. This 40-item instrument evaluates symptom burden and its impact on well-being across eight dimensions. Items are rated on a 5-point Likert scale from 0 (never) to 4 (always), with total scores ranging from 0 to 160..Higher total scores indicate a greater symptom burden. The scale showed high reliability in this study (Cronbach’s α = .947).

#### Family functioning.

The Family APGAR Index [15] was employed. This 5-item questionnaire measures satisfaction with family support across five domains. Items are scored on a 3-point scale (0–2), yielding a total score from 0 to 10. Higher scores indicate better perceived family functioning. The scale exhibited good internal consistency in this study (Cronbach’s α = .875).

### Data analysis

Data analyses were performed using SPSS Statistics version 27.0 and the PROCESS macro version 4.0 for SPSS. To control for potential common method bias, procedural remedies (e.g., anonymity, reverse-scored items) were applied during data collection.

Descriptive statistics were computed for all variables. Continuous variables with a normal distribution are presented as mean ± standard deviation (SD), while those with a skewed distribution are presented as median (interquartile range). Categorical variables are summarized as frequencies and percentages (n, %).

Group differences in symptom experience scores across demographic and clinical characteristics were examined using independent sample t-tests, one-way analysis of variance (ANOVA), or non-parametric tests (Mann-Whitney U, Kruskal-Wallis H) as appropriate. Pearson correlation analysis was conducted to explore the bivariate relationships between resilience, family functioning, and symptom experience.

Hierarchical multiple regression analysis was used to identify factors influencing symptom experience. Variables were selected for the multivariable model based on their theoretical relevance within the Theory of Unpleasant Symptoms (family functioning, resilience) and known clinical confounders (gender, complications), in addition to those showing significant univariate associations (p < 0.05). Variables that showed significant differences in the univariate analyses were entered as covariates in the first block (Model 1). Resilience and family functioning were entered in the second block (Model 2) to examine their unique contribution after controlling for demographic and clinical factors.

Assumption checks for regression analysis. Before conducting the hierarchical regression, we assessed key assumptions. Linearity was examined using partial regression plots. Normality of residuals was assessed using a normal probability plot (P-P plot) and the Shapiro-Wilk test. Homoscedasticity was evaluated by inspecting the scatterplot of standardized residuals versus predicted values. Multicollinearity was tested using variance inflation factors (VIFs). All VIFs were < 2.0, indicating no multicollinearity. Residual plots confirmed acceptable homoscedasticity and approximate normality. No significant influential outliers were detected (Cook’s distance values < 1.0 for all cases).

The mediation hypothesis (that resilience mediates the relationship between family functioning and symptom experience) was tested using PROCESS Macro Model 4 [16,17]. Because this is a cross-sectional study, our mediation analysis tests a statistical indirect effect consistent with the hypothesized TOUS ordering, but it cannot establish temporal precedence or causality. The analysis used 5,000 bootstrap samples to generate bias-corrected 95% confidence intervals (95% BCa CI) for the indirect effect. A confidence interval that did not include zero indicated a statistically significant mediation effect. Significant demographic and clinical covariates identified from the hierarchical regression (gender and complications) were included as control variables in the mediation model.. A two-tailed p-value of less than 0.05 was considered statistically significant for all analyses.

## Results

### Participant characteristics and descriptive statistics

A total of 333 kidney transplant recipients were included in the final analysis. The descriptive statistics for the main study variables are presented in Table 1. The participants reported a moderate level of symptom burden (OTSWI total score: 46.56 ± 23.86). The level of resilience was above average (RS-KTPL-CV total score: 71.46 ± 8.05), with the ‘Positive Attitude’ subscale scoring the highest. Overall, family functioning was at a good level (APGAR total score: 7.49 ± 2.70).

**Table 1:** Scores of symptom experience, resilience, and family functioning (n = 333).

Variable Category	Specific Variable	Mean ± SD	Skewness	Kurtosis
Symptom Experience				
	OTSWI Total Score	46.56 ± 23.86	0.82	1.55
	Sleep problems	5.65 ± 3.08	0.09	−0.52
	Ache of lower limb	5.05 ± 4.29	0.83	0.34
	Fatigue	4.78 ± 3.03	0.31	−0.37
	Cognitive functioning	3.61 ± 1.91	0.12	−0.18
	Basic ADL	1.05 ± 1.99	2.82	9.28
	Mood	2.10 ± 1.94	0.70	−0.13
	Economy	3.07 ± 2.46	0.51	−0.78
	Symptoms	21.27 ± 11.61	0.88	1.99
Resilience				
	RS-KTPL Total Score	71.46 ± 8.05	0.65	−0.50
	Positive Attitude	32.31 ± 4.18	0.59	−0.36
	Medical Management	16.90 ± 2.12	0.32	−1.37
	Social Support	12.87 ± 1.74	0.60	−0.37
	Health Habits	9.38 ± 1.29	0.31	0.27
Family Functioning				
	APGAR Total Score	7.49 ± 2.70	−1.07	0.42
	Adaptation	1.46 ± 0.66	−0.82	−0.43
	Partnership	1.43 ± 0.69	−0.79	−0.55
	Growth	1.50 ± 0.68	−1.02	−0.18
	Affection	1.43 ± 0.69	−0.81	−0.56
	Resolve	1.67 ± 0.58	−1.56	1.39

Note: OTSWI = Organ Transplant Symptom and Well-being Inventory; RS-KTPL = Resilience Scale for Kidney Transplantation; APGAR = Family APGAR Index. A skewness absolute value > 1.0 indicates significant skewness; a kurtosis absolute value > 3.0 indicates abnormal kurtosis. Skewness and kurtosis values indicate that all variables were within acceptable ranges for parametric tests (skewness | < 2|, kurtosis | < 7|).

### Univariate analysis of symptom experience

The results of the univariate analysis (Table 2) showed that symptom experience scores differed significantly based on gender, employment status, place of residence, monthly household income, and the presence of complications (all *p* < .05). Specifically, female patients, those unemployed or with ‘other’ employment status, those residing in rural areas, those with a monthly household income below ¥2000, and those with complications reported significantly higher symptom burden.

**Table 2:** Comparison of symptom experience scores by demographic and clinical characteristics (n = 333).

Variable	Category	n (%)	Symptom Experience Score (Mean ± SD)	Statistic	*p*-value
Gender				*t* = 2.49	0.013
	Male	206	44.03 ± 24.65		
	Female	127	50.67 ± 22.00		
Employment Status				F = 5.25	0.006
	Employed	124	41.35 ± 22.06		
	Retired	61	47.33 ± 21.41		
	Other (e.g., unemployed)	148	50.61 ± 25.53		
Residence				*t* = 2.80	0.005
	Urban	259	44.63 ± 22.95		
	Rural	74	53.35 ± 25.85		
Marital Status				F = 1.38	0.253
	Unmarried	42	45.24 ± 20.31		
	Married	265	46.05 ± 23.40		
	Other (Divorced/Widowed)	26	53.96 ± 32.26		
Monthly Income (¥)				F = 5.89	0.001
	< 2000	69	56.64 ± 30.37		
	2000–5000	103	46.10 ± 20.95		
	5000–10000	87	42.77 ± 22.33		
	> 10000	74	42.28 ± 19.94		
Complications				*t* = 4.00	<0.001
	Yes	87	55.16 ± 25.11		
	No	246	43.52 ± 22.68		

Note: OTSWI = Organ Transplant Symptom and Well-being Inventory; RS-KTPL = Resilience Scale for Kidney Transplantation; APGAR = Family APGAR Index. A skewness absolute value > 1.0 indicates significant skewness; a kurtosis absolute value > 3.0 indicates abnormal kurtosis. All data are presented as group-level statistics (means, frequencies). No individual-level identifiers are included, and cell sizes are sufficiently large to prevent re-identification.

### Correlations among main variables

Pearson correlation analysis (Table 3) revealed that resilience was significantly positively correlated with family functioning (*r* = 0.194, *p* < .01). Both resilience (*r* = −0.222, *p* < .01) and family functioning (*r* = −0.202, *p* < .01) were significantly negatively correlated with the total symptom experience score. These results provided a preliminary foundation for the subsequent mediation analysis.

**Table 3:** Correlations among resilience, family functioning, and symptom experience (n = 333).

Variable	Resilience	Family Functioning	Total Symptom Score
Resilience	1		
Family Functioning	0.194**	1	
Total Symptom Score	−0.222**	−0.202**	1

Note: Values are Pearson correlation coefficients. **p < .01 (2-tailed).

### Factors influencing symptom experience: Hierarchical regression analysis

Hierarchical regression analysis was conducted with the total symptom experience score as the dependent variable. The statistically significant variables identified in the univariate analysis (gender, employment status, residence, monthly income, complications) were entered into the first block (Model 1: demographic and clinical model). Resilience and family functioning were entered into the second block (Model 2: full model). The coding scheme for categorical variables is provided in S2 Table.

Model 1, which included only the demographic and clinical factors, explained 10.9% of the variance in symptom experience (Adjusted *R^2^* = 0.089, *F* = 5.660, *p* < .001). After adding the psychosocial variables (resilience and family functioning) in Model 2, the explanatory power of the model significantly increased to 15.0% (Adjusted *R*^2^ = 0.126), with a significant change in *R*^2^ (Δ*R*^2^ = 0.041, ΔF = 7.79, *p* = .001). This indicates that resilience and family functioning provided unique explanatory power beyond the demographic and clinical variables. The model summary is presented in Table 4.

**Table 4:** Summary of hierarchical regression models for symptom experience (n = 333).

Model	Predictors	R	R^2^	Adjusted R²	ΔR^2^	F	ΔF	p
1	Demographic & Clinical	0.330	0.109	0.089	0.109	5.660	—	<0.001
2	Model 1 + Psychosocial	0.387	0.150	0.126	0.041	6.332	7.85	<0.001

Note: Model 1 predictors: gender, employment status (dummy variables), residence, monthly income (dummy variables), and complications. Model 2 predictors: Model 1 variables plus resilience and family functioning. Dependent variable: OTSWI total score (symptom experience). R = multiple correlation coefficient; R^2^ = coefficient of determination; Adjusted R^2^ = R^2^ adjusted for the number of predictors; ΔR^2^ = change in R^2^ from Model 1 to Model 2; F = F-statistic for the model; ΔF = F-change; p = p-value for the model. Reference categories: employment status = “Other”; monthly income = “5000–10000”.

The final regression model (Table 5) showed that after controlling for demographic factors, the presence of complications (β = 0.169, p = .001) was the strongest positive predictor of a higher symptom burden, indicating that patients with complications reported significantly more symptoms. Both resilience (β = −0.152, p = .005) and family functioning (β = −0.124, p = .022) were significant negative predictors, meaning that higher levels of resilience and better family functioning were associated with lower symptom burden.

**Table 5:** Hierarchical regression coefficients for symptom experience.

Model	Predictor	B	SE	β	t	p	95% CI for B
1	(Constant)	41.514	3.449	—	12.038	<0.001	[34.75, 48.27]
	Gender (Female vs Male)	4.926	2.677	0.100	1.840	0.067	[-0.32, 10.17]
	Employment: Retired vs Other	−4.211	3.181	−0.085	−1.324	0.187	[-10.45, 2.03]
	Employment: Employed vs Other	−2.427	3.558	−0.039	−0.682	0.496	[-9.40, 4.55]
	Residence (Rural vs Urban)	6.186	3.240	0.108	1.909	0.057	[-0.19, 12.56]
	Income: < 2000 vs 5000–10000	10.672	3.964	0.182	2.693	0.007	[2.90, 18.44]
	Income: 2000–5000 vs 5000–10000	0.887	3.460	0.017	0.256	0.798	[-5.89, 7.67]
	Income: > 10000 vs 5000–10000	−0.273	3.618	−0.005	−0.075	0.940	[-7.36, 6.82]
	Complications (Yes vs No)	10.565	2.855	0.195	3.700	<0.001	[4.97, 16.16]
2	(Constant)	83.933	12.298	—	6.825	<0.001	[59.83, 108.04]
	Gender (Female vs Male)	4.081	2.631	0.083	1.551	0.122	[-1.08, 9.24]
	Employment: Retired vs Other	−4.832	3.121	−0.098	−1.548	0.123	[-10.95, 1.29]
	Employment: Employed vs Other	−1.573	3.494	−0.026	−0.450	0.653	[-8.42, 5.27]
	Residence (Rural vs Urban)	5.950	3.178	0.104	1.872	0.062	[-0.30, 12.20]
	Income: < 2000 vs 5000–10000	7.517	3.968	0.128	1.894	0.059	[-0.26, 15.29]
	Income: 2000–5000 vs 5000–10000	−1.067	3.425	−0.021	−0.311	0.756	[-7.78, 5.65]
	Income: > 10000 vs 5000–10000	0.280	3.548	0.005	0.079	0.937	[-6.67, 7.23]
	Complications (Yes vs No)	9.144	2.821	0.169	3.242	0.001	[3.62, 14.67]

Note: CI = confidence interval. Reference groups: gender = male; employment status = “Other”; monthly income = “5000–10000”; complications = “No”. Employment and monthly income were entered as dummy variables. 95% CIs were calculated as B ± 1.96 × SE. Significant predictors (p < .05) are highlighted in bold.

### Mediating role of resilience

Harman’s single-factor test was conducted, and five factors with eigenvalues greater than 1 were extracted. The first factor accounted for 23.70% of the variance, which is below the critical threshold of 40%, indicating that common method bias was not a serious concern in this study. However, Harman’s single-factor test is a relatively conservative indicator; same-source, same-time self-report data may still be subject to common method variance.

The mediation analysis was performed using PROCESS Macro Model 4 (Hayes, 2017), controlling for significant covariates (e.g., gender, complications). Bootstrap analysis with 5000 resamples was applied. The results (see Table 6 and Fig 1) demonstrated:

- Total effect: Family functioning had a significant negative total effect on symptom experience (*B* = −1.789, 95% CI: −2.726 to −0.852).
- Direct effect: The direct effect of family functioning on symptom experience remained significant (*B* = −1.462, 95% CI: −2.402 to −0.523).
- Indirect effect: The indirect effect through resilience (family functioning → resilience → symptom experience) was significant, with a point estimate of −0.326 (Boot *SE* = 0.144, 95% BCa CI: −0.645 to −0.081).

**Table 6:** Analysis of the mediating effect of resilience between family functioning and symptom experience.

Effect Type	Pathway	β	Effect (B)	Boot SE	Boot 95% BCa CI	Effect Ratio (%)
Total Effect	Family → Symptom Experience	−0.202	−1.789	0.476	[−2.726, −0.852]	100%
Direct Effect	Family → Symptom Experience	−0.165	−1.462	0.477	[−2.402, −0.523]	81.6%
Indirect Effect	Family → Resilience → Symptom	−0.037	−0.326	0.144	[−0.645, −0.081]	18.4%

Note: BCa CI = Bias-Corrected and Accelerated Confidence Interval. Unstandardized coefficients (B) are reported. The indirect effect is significant as its 95% BCa CI does not include zero.

**Fig 1:**
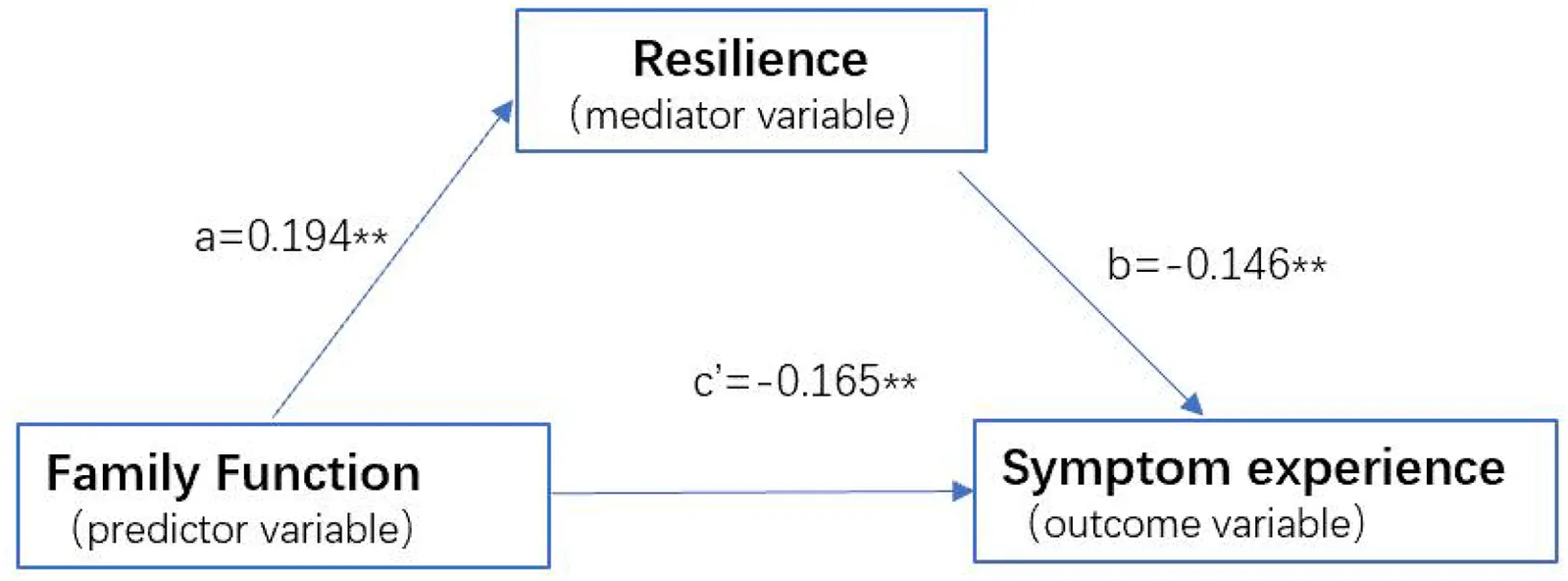
The mediating model of resilience between family functioning and symptom experience. Mediation model of the association between family functioning and symptom experience through resilience. Standardized path coefficients (β) are shown. Path a: family functioning → resilience; Path b: resilience → symptom experience (controlling for family functioning); Path c’: direct effect of family functioning on symptom experience (controlling for resilience). All analyses controlled for significant covariates: gender and complications. **p < .01. Note: This cross-sectional model tests statistical indirect effects consistent with a mediating role but does not establish causality.

Since the bootstrap confidence interval for the indirect effect did not include zero, it indicates that resilience plays a significant partial mediating role in the relationship between family functioning and symptom experience. This indirect effect accounted for 18.4% of the total effect.

## Discussion

### Summary of key findings

Guided by the Theory of Unpleasant Symptoms (TOUS), this study investigated the relationships between family functioning, resilience, and symptom experience in kidney transplant recipients. The key finding of this study is that resilience was consistent with a partial mediating role in the association between family functioning and symptom experience. Specifically, better family functioning was not only directly associated with less severe symptom experience but was also associated with higher levels of resilience, which in turn was associated with reduced symptom burden. This indirect pathway accounted for 18.4% of the total effect, suggesting the potential role of intrapersonal psychological resources in translating external family support into better symptom management. Given the cross-sectional design, these findings should be interpreted as statistical indirect effects consistent with the hypothesized mediation framework, rather than as evidence of causal mediation.

### Interpretation of findings within the context of the literature

#### Predictors of symptom experience.

Our findings confirm that the symptom experience in kidney transplant recipients is multifactorial, influenced by a complex interplay of biomedical and socioeconomic factors. The presence of complications was the strongest predictor of a higher symptom burden, which is consistent with previous research [18]. This underscores the importance of rigorous medical management to prevent and control post-transplant complications.

Furthermore, socioeconomic factors play a significant role. Patients with the lowest monthly household income (<¥2000) reported the highest symptom burden. Financial hardship can limit access to self-paid medications, nutritional support, and timely follow-up visits, potentially leading to poorer medication adherence and disease control, thereby potentially contributing to a pattern of “poverty-worsening symptoms” [14,19–21]. The significant disparity in symptom burden between rural and urban residents also points to structural barriers, such as difficulties in accessing specialized follow-up care and transportation challenges, which can delay the identification and management of emerging problems [22,23].

The observed gender difference, with women reporting a higher symptom burden, aligns with findings in chronic illness populations [24]. Beyond potential biological factors, this disparity may be influenced by gendered sociocultural roles. Women often take on the role of primary family caregiver even while managing their own illness, which may contribute to role overload and self-neglect, potentially exacerbating symptom perception and reporting [25,26]. Furthermore, gender socialization may encourage women to be more attentive to bodily sensations and more willing to report symptoms. We also note that the intersection of multiple disadvantaged identities, such as being female and having a low income, may compound this burden.

#### The protective roles of family functioning and resilience.

Both correlation and regression analyses consistently demonstrated that higher family functioning and greater resilience were independent correlates of lower symptom burden. This suggests that internal psychological resources and external support systems may constitute effective, complementary mechanisms for counteracting the symptom burden.

Resilience may exert its protective effect through multiple dimensions measured by the RS-KTPL-CV: a *positive attitude* may facilitate cognitive reframing of the illness experience [27]; effective *medical management* may enhance medication adherence and self-monitoring [28]; and healthy *habits* improve behaviours like sleep and exercise, thereby potentially alleviating physical symptoms like fatigue [29]. Concurrently, family functioning may provide practical support (e.g., medication reminders, accompaniment to appointments) that may improve treatment adherence, and emotional support that may enhance psychosocial adaptation [30].

#### The mediating role of resilience.

The core contribution of this study is the empirical demonstration of an indirect effect consistent with resilience as a partial mediator. This finding suggests a psychological mechanism through which the benefits of family support may be translated into a reduced symptom experience. We hypothesize that a supportive family environment, characterized by low conflict and high emotional warmth, may be associated with the development and maintenance of psychological resilience by enhancing an individual’s sense of self-efficacy and providing a context for developing adaptive coping strategies [31]. This higher level of resilience then may act as a personal resource, enabling individuals to more effectively appraise and manage illness-related stressors, employ problem-focused coping, and regulate emotions, thereby potentially reducing the perceived intensity, distress, and interference of symptoms [5,32,33]. This indirect pathway may help explain the counterintuitive scenario where patients with ostensibly good family support still experience poor outcomes—their internal psychological resources (resilience) may be insufficient to translate external support into tangible health benefits. We also acknowledge the plausible reverse pathway whereby higher symptom burden may erode resilience and family cohesion over time, which cannot be tested with cross-sectional data.

#### Modest explanatory power of the model.

Our final model explained only 15% of the variance in symptom experience (Adjusted R^2^ = 0.126). This indicates that the majority of symptom burden is determined by factors not captured in our study, such as biomedical parameters (e.g., graft function, infection status, immunosuppression levels), other psychosocial variables (e.g., coping styles, health literacy, social support beyond the family), and healthcare system factors (e.g., access to care, medication costs). Therefore, while resilience and family functioning are meaningful contributors, they are part of a broader set of determinants.

### Implications for practice and research

The findings suggest a shift towards a biopsychosocial model in post-transplant care. To identify patients at high risk for severe symptom burden, routine assessment of both family functioning and resilience may be essential. We propose a tiered intervention strategy as a hypothesis for future testing:

1. Systematic Screening: Integrate brief tools (e.g., Family APGAR, RS-KTPL-CV) into routine follow-ups at key post-transplant intervals to identify patients needing psychosocial support.
2. Targeted Interventions:
  ◦ For those with poor family functioning, offer family-centered consultations to enhance communication, role negotiation, and conflict resolution.
  ◦ For patients with low resilience, provide structured training focusing on cognitive reframing, stress management, and strength-building, deliverable via group sessions or digital platforms.
3. Integrated Care: Develop pathways that concurrently address family support and individual resilience. such a dual-path approach may ensure that external resources are effectively translated into internal capacity, potentially mitigating symptoms through synergistic effects.

### Limitations and future directions

This study has several limitations. First, its cross-sectional design precludes definitive conclusions about causality. Therefore, longitudinal studies are needed to verify the temporal and causal relationships that are consistent with our mediation model. Second, the use of a convenience sample from a single centre in Wuhan, China, over a short two-month period may limit the generalizability of the findings. Participants were required to be medically stable and free from major comorbidities or psychiatric disorders, which likely excluded more vulnerable transplant recipients. The mean OTSWI score was relatively low (46.56/160), indicating a floor effect; this further limits generalizability to patients with higher symptom burden. Future research should employ multi-centre designs with larger, more diverse samples to enhance the external validity of our conclusions. Third, data based on self-report measures are susceptible to subjective bias and common method variance. Although Harman’s single-factor test did not indicate severe bias, this test is relatively weak. Future studies could benefit from incorporating objective measures (e.g., electronic medication adherence monitors, clinical biomarkers of symptom burden) to complement patient-reported outcomes. Fourth, we did not measure key clinical confounders such as eGFR, immunosuppressive regimen details, or donor type. Fifth, the exclusion of participants with psychiatric disorders may have restricted variability in resilience and symptom experience, potentially underestimating the true associations.

The present cohort partially overlaps with the sample reported in our prior methodological study (Tang et al., 2025) [13], which focused on the validation of the RS-KTPL instrument. Because the two studies recruited from the same outpatient clinic at different time periods, partial participant overlap cannot be excluded. However, the two studies address different research questions and report different outcomes.

## Conclusion

This study demonstrates that the findings are consistent with resilience partially mediating the association between family functioning and symptom experience in kidney transplant recipients. Better family functioning is associated with higher resilience, which in turn is associated with less severe symptom burden. These findings underscore the potential importance of assessing both family support systems and individual psychological resources, such as resilience, in clinical practice. Developing and implementing integrated interventions that target both the family environment and patients’ resilience skills may hold promise for potentially alleviating the symptom experience and improving the overall well-being of individuals living with a kidney transplant. However, given the cross-sectional design and modest explanatory power, future longitudinal and interventional research is needed to confirm these associations and test their clinical utility.

## Supporting information

S1 Checklist STROBE checklist for cross-sectional studies. (DOC)

S2 Table Coding scheme for categorical variables used in hierarchical regression analysis. (DOCX)
