# Supplementary material for: Resilience and its mediating role in the association between family functioning and symptom experience in kidney transplant recipients: A cross-sectional study
Source: PLoS One. 2026 Nov 12;21(11):e0361968. doi: 10.1371/journal.pone.0361968 (PMC13759052; doi:10.1371/journal.pone.0361968)
Supplement: Supplementary file 1 [file pone.0361968.s002.docx]

**Supplementary Table S2. Coding scheme for categorical variables used in hierarchical regression analysis**

| **Variable** | **Category** | **Dummy variable(s)** | **Reference group** |
| --- | --- | --- | --- |
| **Gender** | Male | — | Male |
|  | Female | Female = 1, Male = 0 |  |
| **Employment status** | Employed | — | Employed |
|  | Retired | Retired = 1, otherwise 0 |  |
|  | Other (e.g., unemployed) | Other = 1, otherwise 0 |  |
| **Residence** | Urban | — | Urban |
|  | Rural | Rural = 1, Urban = 0 |  |
| **Monthly income (¥)** | < 2000 | — | < 2000 |
|  | 2000–5000 | Income1 = 1, otherwise 0 |  |
|  | 5000–10000 | Income2 = 1, otherwise 0 |  |
|  | > 10000 | Income3 = 1, otherwise 0 |  |
| **Complications** | No | — | No |
|  | Yes | Yes = 1, No = 0 |  |

*Note: Note: Employment status was coded with “Employed” as the reference category, creating two dummy variables (Retired, Other). Monthly income was coded with “<2000” as the reference category, creating three dummy variables (2000–5000, 5000–10000, >10000). Gender, residence, and complications were entered as single dummy variables. All dummy variables were entered simultaneously into the hierarchical regression models (Model 1 and Model 2).*
