# Supplementary material for: Resilience and its mediating role in the association between family functioning and symptom experience in kidney transplant recipients: A cross-sectional study
Source: PLoS One. 2026 Nov 12;21(11):e0361968. doi: 10.1371/journal.pone.0361968 (PMC13759052; doi:10.1371/journal.pone.0361968)
Supplement: Supplementary file 2 [file pone.0361968.s003.docx]

**Reviewer Report**

**Manuscript title**

**Resilience mediates the relationship between family functioning and symptom experience in kidney transplant recipients: A cross-sectional study**

**Overall recommendation**

**Major Revision**

There are several concerns that need to be addressed before the manuscript can be considered for publication.

Most importantly, there are internal inconsistencies in the statistical reporting, especially the direction/sign of key regression coefficients, and the manuscript at times overstates causal or mediational inference despite the cross-sectional design.

In addition, the ethics reporting in the submission materials appears inconsistent with the manuscript text, and the data availability statement may not comply with journal policy. Clarification and, if needed, correction of these issues are essential.

**Major comments**

**1. Internal inconsistency in regression signs and interpretation**

The manuscript contains major inconsistencies in the reporting of regression findings. In the abstract, resilience is described as an independent predictor with **β = 0.146, p = .007**, whereas Table 6 reports **B = -0.433, β = -0.146, p = .007**, which implies the opposite direction. Likewise, the text states that complications were the strongest positive predictor of higher symptom burden, yet Table 6 shows **B = -9.534, β = -0.176** for “Complications (Yes vs No),” despite the univariate results showing that participants with complications had markedly higher symptom scores than those without complications. These discrepancies make the current results difficult to interpret and undermine confidence in the analysis. The authors should carefully review the coding of variables, revise the tables and text for internal consistency, and ensure that the abstract, results, discussion, and figure all reflect the same correctly interpreted model outputs.

**2. Mediation language is too strong for a cross-sectional design**

The manuscript repeatedly states that resilience “mediates” the relationship between family functioning and symptom experience. While indirect effects can be estimated in cross-sectional data, temporal ordering and causality cannot be established from a single time point. Although the limitation is briefly acknowledged later, the title, abstract, and discussion still frame the findings too definitively. The manuscript should be revised to use more cautious language, such as “an indirect effect consistent with mediation” or “resilience may function as an intermediary pathway.” This issue is especially important because the central claim of the paper depends on temporal and conceptual ordering that the design cannot prove.

**3. Ethics reporting is inconsistent**

The submission material lists the ethics statement as **“N/A”**, even though this is clearly a human participant study, and the manuscript itself states that approval was obtained from the Medical Ethics Committee and that electronic informed consent was secured from all participants. This inconsistency must be corrected in the submission system and harmonized with the manuscript. Human-subject research requires complete and accurate ethics reporting.

**4. Data availability statement may not meet journal's requirements**

The manuscript states that the data are not publicly available due to privacy concerns and are available from the corresponding author upon reasonable request and ethics committee approval. However, the submission pages also note that “data available on request” is generally not sufficient. The authors should provide a data availability statement that conforms to journal policy, ideally by depositing a de-identified dataset or by specifying a formal institutional access process rather than relying on author-mediated requests alone.

**5. Clarify coding and treatment of categorical predictors**

Table 4 indicates that employment status was coded as **0, 1, 2** and monthly income as **0, 1, 2, 3**. If these were entered into regression as single numeric variables, this imposes an ordinal linear assumption that may not be justified, especially for employment status. The manuscript should explicitly state how categorical variables were entered into the regression model and whether dummy coding was used. If not, the regression should be reconsidered and potentially re-run using appropriate categorical coding.

**6. More detail is needed on regression assumptions and analytic diagnostics**

The methods describe descriptive analyses, group comparisons, correlations, and regression, but key diagnostics for hierarchical regression are not reported. The authors should state whether assumptions of linearity, normality of residuals, homoscedasticity, independence, and multicollinearity were checked. Given that some variables showed skewness or kurtosis, it would also be helpful to clarify whether the regression residuals were approximately normal and whether any influential observations were detected.

**7. Common method bias is not fully addressed**

The manuscript states that common method bias was assessed using Harman’s single-factor test and concludes that it was not a major concern. Because all main variables were collected by self-report at the same time, this issue is important. Harman’s test alone is generally considered a weak approach. The authors should either temper their claim and acknowledge that common method variance may still be present, or provide a stronger rationale for why the findings are unlikely to be substantially inflated by shared method variance.

**8. The discussion should more clearly acknowledge the modest explanatory power of the model**

The final model explained **14.2%** of the variance in symptom experience, with an adjusted R² of **0.123**. This suggests the model captures only a limited proportion of symptom burden. The authors should interpret their findings more cautiously and acknowledge that substantial variance remains unexplained, likely due to other biomedical, psychosocial, and treatment-related factors not included in the model.

**9. Sample size justification should be strengthened**

The sample size rationale is based on a generic rule of 5–10 participants per variable. Because mediation is a main analytical aim, a more appropriate power rationale for detecting indirect effects would strengthen the manuscript. At minimum, the authors should clarify why this approach was considered adequate for both hierarchical regression and mediation analysis.

**10. Potential conceptual overlap between predictor and mediator should be discussed**

The resilience scale used in the study includes a “social support” dimension, while family functioning is also a central independent variable. This raises the possibility of conceptual overlap between the predictor and mediator, which could partly inflate associations. The authors should discuss this issue and explain why the constructs are still considered sufficiently distinct for the proposed mediation model.

**Minor comments**

**1. Title and wording**

The title may overstate the claim by using definitive mediation language in a cross-sectional study. A more cautious title would be preferable.

**2. Abstract**

Please correct the sign of the resilience coefficient in the abstract and ensure consistency with the final regression table. Also, the conclusion should avoid causal implications stronger than the design permits.

**3. Methods**

Please provide more detail on the clinical variable “complications,” including whether this was self-reported or medically verified and what types of complications were included. Also clarify the exact attention-check item and the criteria for identifying a straight-line response pattern.

**4. Results presentation**

Table 6 appears without a fully clear title in the running text, and the coefficients table should be more explicitly labeled as Model 1 and Model 2 regression coefficients for symptom experience. Also confirm whether the coefficients shown in Figure 1 are standardized or unstandardized, because the caption states unstandardized coefficients while the displayed path values resemble standardized coefficients.

**5. Discussion**

Some interpretive statements are somewhat speculative, especially those related to gender roles and compounded disadvantage. These may be reasonable hypotheses, but they should either be more carefully supported by the cited literature or toned down.

**6. Repetition**

The section “Relevance to clinical practice and future research” largely repeats points already made under “Implications for practice and research” and “Limitations and future directions.” Condensing these sections would improve flow and reduce redundancy.

**7. Language and formatting**

A careful language edit is recommended. There are a few punctuation and formatting issues, such as double punctuation in the methods section and occasional spacing inconsistencies in citations and notation.

**Comments to the editor**

The study may ultimately be publishable, but at present it requires **major revision** because of important internal inconsistencies in the statistical reporting and interpretation. In particular, the regression signs for resilience and complications appear contradictory across sections, and the mediation claim is stronger than warranted by a cross-sectional design. In addition, the ethics reporting in the submission materials is inconsistent with the manuscript, and the data availability statement may not satisfy journal requirements. I recommend asking the authors to revise substantially and resubmit with corrected analyses/reporting and more cautious interpretation.
