# Supplementary material for: Resilience and its mediating role in the association between family functioning and symptom experience in kidney transplant recipients: A cross-sectional study
Source: PLoS One. 2026 Nov 12;21(11):e0361968. doi: 10.1371/journal.pone.0361968 (PMC13759052; doi:10.1371/journal.pone.0361968)
Supplement: Supplementary file 3 [file pone.0361968.s004.docx]

**Title:** Resilience mediates the relationship between family functioning and symptom experience in kidney transplant recipients: A cross-sectional study

**Overall Comments**

This manuscript describes an important and clinically relevant topic related to psychosocial factors and symptom burden among post-kidney transplant patients. The study is well written with the use of validated assessment tools. However, some minor but important discrepancies and methodological issues need to be addressed before acceptance.

**Abstract**

1. In the Results section of the abstract (Lines 25-26), resilience is reported as a positive predictor (β = -0.146), whereas Table 6 reports a negative predictor for resilience (β = -0.146). Please clarify and correct this inconsistency.

**Introduction**

2. The introduction provides a good overview of symptom burden after kidney transplantation and the possible role of family functioning and resilience. But the statement “Family functioning, as a crucial component of social support, plays a pivotal role in influencing patient outcomes” in line 45 needs a reference.

**Methods**

3. The exclusion criteria related to “stressful life events” (line 88) are unclear and need further clarity on the types of stress events.
4. The sample size calculation was done based on 5-10 participants per independent variable for regression analysis (lines 90-96). Additional justification regarding statistical power for total sample size can strengthen the method (final number of participants is either 5 or 10 per independent or any statistical formula).
5. As you mentioned, a total of 335 questionnaires were distributed, with two invalid responses; 333 participants were included in the final analysis. Any denial from eligible participants?
6. Important transplant-related clinical variables such as graft function or rejection history after surgery are missing.
7. Harman’s single factor test to assess common method variance parts needs to be described in more detail for better clarity.

**Results**

8. Line 213: Please add table 6 in the heading.
9. There is an inconsistency regarding the complications variable in the regression analysis. Table 6 shows a negative regression coefficient for complications. This conflicts the univariate findings (line 175) and the interpretation provided in the discussion (line 259). Please recheck the reporting error.

**Discussion**

10. The discussion section is well written and relevant. But I am confused about some interpretations related to low income in lines 262-266, as monthly income was no longer significant in the final regression model (table 6 model 2).
11. Line 266: Check the citation numbers issue
12. I think some explanations related to syndemic disadvantage (line 277) are not directly supported by the result.
13. Line 302: Check the citation numbers issue
