# Supplementary material for: Resilience and its mediating role in the association between family functioning and symptom experience in kidney transplant recipients: A cross-sectional study
Source: PLoS One. 2026 Nov 12;21(11):e0361968. doi: 10.1371/journal.pone.0361968 (PMC13759052; doi:10.1371/journal.pone.0361968)
Supplement: Supplementary file 4 [file pone.0361968.s005.docx]

Dear Editor,

Thank you for the opportunity to review the manuscript titled “Resilience mediates the relationship between family functioning and symptom experience in kidney transplant recipients: A cross-sectional study.”

The manuscript examines whether resilience mediates the association between family functioning and symptom experience among adult kidney transplant recipients, using validated Chinese versions of the RS‑KTPL, OTSWI, and Family APGAR, and PROCESS mediation analysis. The research question is timely and clinically meaningful, and the theoretical anchoring in the Theory of Unpleasant Symptoms (TOUS) is appropriate. However, several important methodological and reporting issues require attention before the findings can be interpreted with confidence.

Major comments:

1. The design is cross‑sectional, yet the mediation model is described in strongly causal language (e.g., “resilience mediates,” “effect of family functioning on symptom experience,” “pathway through which family support translates into outcomes”). With simultaneous measurement, the temporal ordering of family functioning → resilience → symptom experience cannot be established.

Although this limitation is mentioned in the discussion, the causal language remains strong. I recommend softening the wording throughout (title, abstract, methods, results, discussion, conclusion) to emphasize “associations,” “indirect statistical pathways,” or “theoretical mediation” rather than causal effects.

It would also be helpful to explicitly state in the methods and discussion that, because the study is cross‑sectional, mediation should be interpreted as a statistical indirect association consistent with the hypothesized TOUS causal ordering framework rather than as evidence of causal mediation.

2. The authors acknowledge the limitation in external validity, but the implications are underdeveloped. There is minimal description of the transplant program context (e.g., typical time post‑transplant, standard immunosuppression protocol at the center, follow‑up schedule, and usual psychosocial support). This makes it difficult for readers to evaluate the generalizability of the findings.

Please add more detail about the clinical setting and care pathways (median/mean time since transplant, standard follow‑up structure, presence and type of formal psychosocial services). In addition, please discuss how single-center convenience sampling and local health‑system characteristics may shape resilience, family functioning, and symptom burden, and be more explicit about the populations to which these results may or may not generalize.

Because participants with psychiatric disorders were excluded from the study sample, the findings may underestimate the relationship between resilience, family functioning, and symptom burden in broader transplant populations. Please discuss how this exclusion criterion may affect external validity and whether psychosocial screening practices at your center differ from those used elsewhere.

3. Sample size was justified using a conventional rule-of-thumb approach, “5–10 participants per independent variable” rule. The text suggests that 17 variables were planned, but it is not entirely clear which variables these are or whether the final models match this plan. Please list all variables included in this calculation and confirm that the final regression and mediation models align with the original analytic plan. If any variables were dropped or added post‑hoc, this should be clearly described and justified.
4. The models control for several socio‑demographic and clinical factors (gender, employment, residence, income, complications), but important potential confounders such as time since transplant, graft function, comorbid mental health diagnoses, and immunosuppressive regimen are not included.

Time since transplant is likely to influence both resilience and symptom experience, and complications are coded as a simple binary variable, which may oversimplify clinical status. Please clarify which clinical variables were collected (e.g., time since transplant, type/number of complications, psychiatric history, graft function indicators) and justify their inclusion or exclusion in the multivariable and mediation models. If possible, sensitivity analyses including key clinical variables (such as time since transplant) would be informative, or at least a statement on whether including them materially changes the estimates for resilience and family functioning.

Regarding the inclusion criterion of “at least 3 months post‑transplant”: Please clarify the rationale for selecting ≥3 months post-transplant as an inclusion criterion and discuss whether symptom burden and resilience may differ during earlier post-transplant phases.

5. The authors rely on Pearson correlations and linear regression, but provide limited information on how assumptions (normality, linearity, homoscedasticity, and multicollinearity) were assessed. Although skewness and kurtosis are reported, they are not clearly linked to the regression assumptions. I recommend briefly describing how regression assumptions were checked (e.g., inspection of residual plots, tests for multicollinearity such as VIFs) and whether any transformations or robust procedures were considered.
6. The indirect effect accounted for 18.4% of the total effect, but the clinical meaning of the effect sizes is not discussed. For instance, what does a 1‑point increase in APGAR or RS‑KTPL correspond to in terms of change in OTSWI score, and is that difference clinically meaningful?

The paper describes a “core contribution” to understanding mechanisms, but the associations between resilience/family functioning and symptom burden are modest, and the model explains only about 14% of the variance in symptoms. The discussion occasionally overstates the model's explanatory contribution, given the modest variance explained. It would be helpful to discuss the practical significance of these effect sizes (e.g., how they compare to known minimal clinically important differences, if available), and to adopt a more modest tone about the explanatory power of the model, emphasizing that resilience and family functioning are part of a broader set of determinants.

Minor comments:

1. Title and abstract: Consider softening the title to reflect association rather than confirmed mediation in a causal sense (e.g., “Resilience and its mediating role in the association between family functioning and symptom experience…”).

In the abstract, please explicitly state that this was a single‑center, convenience sample with a cross‑sectional design, and avoid causal phrasing such as “predictors” or “effect” where not warranted.

2. Introduction: The “paradox” of patients with good family support but poor outcomes is an interesting point; consider citing empirical work in transplantation or similar chronic illness populations that documents this phenomenon.
3. Methods – participants: Clarify the definition and assessment of “major comorbidities or stressful life events” used as exclusion criteria (how were these identified, by whom, and over what time frame?).

Provide more detail on recruitment, including the number of patients approached, the proportion who declined, and the reasons for non‑participation if available.

4. Methods – measures: For each instrument (RS‑KTPL‑CV, OTSWI, APGAR), briefly summarize, where possible, cut‑points indicating low/medium/high levels to help readers interpret the descriptive statistics.

Describe how any reverse‑scored items were handled.

5. Results – descriptive and univariate analyses:

For Table 1, it would be more informative if it presented at first the sample’s socio‑demographic and clinical characteristics (age, sex, income, residence, time since transplant, complications, etc.) and symptom, resilience, and family functioning scores presented in separate table of number 2. Currently, it is difficult to obtain a quick overview of the sample.

You do not need to list skewness and kurtosis for every variable in the table. Instead, state in the methods or results that all key variables showed acceptable skewness and kurtosis, and that the assumptions of approximate normality were met, rather than cluttering the table with these indices.

In Table 2, some note text appears duplicated or misaligned (e.g., RS‑KTPL and APGAR are mentioned in the notes to a table that only shows symptom variables). Please revise table notes so they refer only to what is presented.

6. Results – regression and mediation tables:

In Table 2, it is unclear why “t” and “F” are surrounded by asterisks. Standard practice is to label test statistics simply as t or F, with asterisks used next to p‑values to indicate significance levels. Please clarify and standardize this notation.

In Table 5, several abbreviations (R, R², ΔR², F) are not fully defined. Please spell out what each abbreviation stands for in the table footnote (e.g., R = correlation coefficient; R² = coefficient of determination; ΔR² = change in R²) and explicitly report ΔR² and the F‑change, as these are central to the hierarchical regression.

Table 4 (“Assignment of variables for hierarchical regression”) is essentially methodological. It would improve the flow to move this table (or its contents) to the Methods section or to the supplementary material.

In the text (around line 209), you refer to “the final regression model” but didn’t write “Table 6” in the line of the title of this table.

There appears to be a sign inconsistency between the description in the text and the coefficients for complications in Table 6 (B is negative but is described as a “positive predictor”). Please double-check coding and ensure that the interpretation is clearly explained.

7. Figures and causal structure:

The mediation figure (Figure 1) is helpful; please ensure that all covariates included in the model are listed in the figure legend and consistent with the text.

Because you are testing a mediation model and making assumptions about causal ordering among family functioning, resilience, and symptom experience, including a directed acyclic graph (DAG) or a similar conceptual diagram may improve transparency and strengthen the paper. This could clarify which variables are considered exposures, mediators, outcomes, and confounders.

8. Discussion:

The discussion is rich and engages with the broader literature, but is somewhat long and occasionally speculative, particularly when introducing concepts such as “syndemic” and “secure base”. A more concise discussion, more tightly linked to the actual findings, would improve readability.

Excluding participants with psychiatric disorders may have restricted variability in resilience and symptom experience and may limit applicability to broader transplant populations, in whom anxiety and depression are common.

Overall, this manuscript addresses an important and underexplored aspect of post‑transplant care and uses appropriate, validated instruments and mediation analysis. However, the cross-sectional nature of the data, relatively modest effect sizes, limited control for key clinical confounders, and, at times, overly causal interpretations of mediation warrant substantial revision. With attention to these issues—particularly clarifying the design's causal limitations, improving the description and handling of covariates, and tightening the discussion—the paper could make a useful contribution. I recommend a major revision.
