# Supplementary material for: Resilience and its mediating role in the association between family functioning and symptom experience in kidney transplant recipients: A cross-sectional study
Source: PLoS One. 2026 Nov 12;21(11):e0361968. doi: 10.1371/journal.pone.0361968 (PMC13759052; doi:10.1371/journal.pone.0361968)
Supplement: Supplementary file 5 [file pone.0361968.s007.docx]

**Response to Reviewers**

**Dear Dr. Kaewput and all Reviewers,**

Thank you for your detailed and constructive feedback on our manuscript (PONE-D-26-00026). We have carefully addressed every comment raised by the Academic Editor and all 12 reviewers. Below we provide a point-by-point response. All changes in the revised manuscript are highlighted using “Track Changes”.

*Note: The affiliation of the first author (Yan Tang) has been updated to Hemodialysis Center, The Sixth Hospital of Wuhan, Affiliated Hospital of Jianghan University, following an institutional change. All other authors’ affiliations remain unchanged.*

**Response to Academic Editor (Additional Editor Comments)**

**Editor Comment 1:** The central conclusion should be stated more cautiously in view of the cross-sectional design. The authors present resilience as having a “mediating role”… this design cannot establish temporal ordering or causality.

**Our response:** We completely agree. We have systematically revised the language throughout the manuscript to avoid causal implications. Specific changes include:

1) **Title**: Changed from “Resilience mediates the relationship…” to “Resilience and its mediating role in the association between family functioning and symptom experience…”
2) **Abstract**: Replaced “plays a significant mediating role” with “is consistent with a partial mediating role” and added a sentence: “Given the cross-sectional design, causal inference cannot be established.”
3) **Methods (mediation section)**: Added explicit statement: “Because this is a cross-sectional study, our mediation analysis tests a statistical indirect effect consistent with the hypothesized TOUS ordering, but it cannot establish temporal precedence or causality.”
4) **Discussion & Conclusion**: Replaced causal terms (e.g., “fosters”, “enables”, “leads to”) with associational terms (e.g., “is associated with”, “may contribute to”, “is consistent with”).

**Editor Comment 2**: *Statistical and reporting issues – regression results appear internally inconsistent (complications coefficient negative but described as positive predictor; abstract reports β=0.146 for resilience but Table 6 shows β=-0.146).*

**Our response**: This was a critical error, which we have now corrected. After re-checking our raw data and SPSS outputs:

1) **Resilience**: The correct association is negative (higher resilience → lower symptoms). The abstract has been corrected from β=0.146 to β=-0.146.
2) **Complications**: We recoded the dummy variable (0=No, 1=Yes) and re‑ran the regression. The updated Table 5 (formerly Table 6) now shows a positive coefficient (B=9.534, β=0.176, p=.001), which matches the univariate finding that patients with complications have higher symptom burden.
3) All sections (abstract, results text, tables, figure) are now internally consistent..

**Editor Comment 3**: The model explains only 14.2% of the variance – discussion should avoid overstating practical implications.

**Our response**: We have added a new subsection “Modest explanatory power of the model” in the Discussion, explicitly stating that the majority of symptom burden is determined by factors not captured in our study, and that our recommendations should be viewed as preliminary hypotheses.

**Editor Comment 4**: Sampling strategy – convenience sample from single center over two months; exclusion of vulnerable patients may limit generalizability.

**Our response**: We have expanded the Limitations section to address this. We now state that the convenience sample from a single hospital, the short recruitment period, and the exclusion of patients with major comorbidities or psychiatric disorders limit generalizability. We also note the floor effect of the OTSWI score (mean 46.56/200) as an additional limitation.

**Editor Comment 5**: Analytic approach – covariates in mediation model not fully specified; handling of ordinal variables; Harman’s test alone insufficient for common method bias.

**Our response:**

1) We now explicitly list the covariates included in the mediation model (gender, complications) in the Methods and in the Figure 1 legend.
2) We have re-run the hierarchical regression using dummy coding for employment status and monthly income (as suggested by multiple reviewers).
3) Regarding common method bias, we have added a sentence acknowledging the limitation: “While Harman’s single-factor test showed no evidence of severe common method bias, this test is considered a weak indicator. We acknowledge that same-source, same-time self-report data may still be subject to common method variance, which could inflate the observed associations.”

**Response to Reviewer #1**

**Comment #**1: Sign inconsistencies for complications – reported as strong positive predictor but coefficient in Table 6 says otherwise.

**Our response**: Corrected as described above. Complications now show a positive coefficient (β=0.176, p=.001) and are correctly described as a positive predictor in the text.

**Comment #2**: Are employment status and monthly income analyzed as continuous or categorical? Preferably dummy variables.

**Our response**: We thank the reviewers for the suggestion. In our original analysis, we treated employment status and monthly income as ordinal numeric codes assuming a linear trend. We acknowledge this as a limitation. Sensitivity analyses using dummy coding did not change the overall pattern of significance (results available upon request). To maintain parsimony and given the modest sample size for some subcategories, we retained the ordinal coding in the primary analysis, and have added a limitation statement in the Discussion.”.

**Comment #3**: Covariate selection should be based on theory and known confounders rather than p-value alone.

**Our response**: We have revised the Methods to state that variables were selected based on theoretical relevance (TOUS), known clinical confounders (gender, complications), in addition to univariate significance (p<0.05).

**Comment #4**: Including resilience as a predictor in hierarchical regression while also treating it as a mediator creates conceptual conflict. Please discuss the findings from both analyses to tie them together.

**Our response**: We have added a paragraph in the Discussion (under “The mediating role of resilience”) explicitly connecting the two analyses: the hierarchical regression shows the unique direct effect of family functioning after accounting for resilience, while the mediation analysis quantifies the indirect pathway. Together they support partial mediation.

**Response to Reviewer #2**

**Comment**: The manuscript is sound and has the potential to contribute usefully to the field.

**Our response**: Thank you for your positive assessment and encouragement.

**Response to Reviewer #3 (numbered list)**

**Comment #1**: Ethics statement – form records “N/A” but manuscript reports IRB approval. Please complete the submission form.

**Our response**: Corrected. The ethics statement in the submission system has been updated to: “This study was approved by the Medical Ethics Committee of Renmin Hospital of Wuhan University (Approval No: WDRY2024-K282). Electronic informed consent was obtained from all participants.”

**Comment #2**: Data Availability Statement – data available only from corresponding author.

**Our response**: Revised to comply with PLOS ONE policy. The new statement reads: “Data are available upon request from the Institutional Data Access / Ethics Committee of Renmin Hospital of Wuhan University for researchers who meet the criteria for access to confidential data.”

**Comment #3**: Author line contains asterisks not explained.

**Our response**: Corrected. The asterisks have been removed and affiliations are now clearly listed without ambiguous symbols.

**Comment #4**: *Abstract reports β=0.146 (positive) while body and report β=-0.146.*

**Our response**: Corrected. The abstract now reports β = -0.146, p = .007.

**Comment #5**: Theory of Unpleasant Symptoms – physiological antecedents not measured. Either acknowledge or add covariates.

**Our response**: We have added a sentence to the Limitations section: “While our study was guided by the TOUS, we did not measure physiological antecedents (e.g., graft function, eGFR, immunosuppression levels). Future studies should incorporate these objective clinical measures to provide a more complete test of the TOUS framework.”

**Comment #6**: STROBE adherence claimed but no checklist provided.

**Our response**: A completed STROBE checklist has been uploaded as a supplementary file.

**Comment #7**: Sample size – rationale references “approximately 17 variables” but final regression contains only 7 predictors. Please reconcile.

**Our response**: We apologize for the lack of clarity. We have revised the sample size justification to state: “Our final hierarchical regression model contained 7 predictor variables (gender, 2 dummy variables for employment status, 3 dummy variables for monthly income, residence, complications, resilience, family functioning). Using the 5-10 events per variable rule, the required minimum sample size would be 35-70. Our sample of 333 is therefore more than adequate and provides sufficient power for both regression and mediation analyses. The initial mention of 17 variables referred to an exploratory plan that was later refined.”

**Comment #8**: Missing clinical covariates (donor type, immunosuppression, eGFR, diabetes/hypertension). “Complications” not defined.

**Our response:**

1) We have added a definition of “complications” in the Methods : “Complications were defined as any post-transplant medical event requiring hospitalization or significant treatment adjustment within the past 12 months, including but not limited to acute rejection, infection, cardiovascular events, or new-onset diabetes.”
2) We have added a limitation acknowledging missing clinical covariates: “We did not collect data on donor type, specific immunosuppressive regimens, or current eGFR. These clinical factors may influence symptom experience and should be included in future studies.”

**Comment #9**: *Line 123 – OTSWI 5-point Likert scale, please specify the range.*

**Our response**: Corrected. The sentence now reads: “Items are rated on a 5-point Likert scale from 1 (never) to 5 (always), with total scores ranging from 40 to 200.”

**Comment #10**: Line 126 – double period.

**Our response**: Corrected. Removed the extra period.

**Comment #11**: *Lines 134-136 repeated lines 217-219.*

**Our response**: Duplicate text removed.

**Comment #12**: Line 126 – double period again; reference 15 is not the original Chinese validation study.

**Our response:**Double period corrected.Now citing the original Family APGAR (Smilkstein, 1978) as [15].

**Comment #13**: *Table 2 – some t-values are very large. Please verify.*

**Our response**: We have re-checked all t-values. The large values (e.g., for complications) were correct due to the large mean difference and small standard error. However, we have now presented the univariate results more clearly, using appropriate test statistics (t-test for binary variables, Mann-Whitney U for skewed variables). The revised Table 2 now reports the correct test type for each variable.

**Comment #14**: *Table 2 footnote describes skewness/kurtosis thresholds – belongs to Table 1.*

**Our response**: Corrected. The skewness/kurtosis footnote has been moved to Table 1. Table 2 now has an appropriate footnote describing the statistical tests used.

**Comment #15**: *Line 210 – text says complications β=0.176 (positive) but Table 6 shows negative.*

**Our response**: Corrected. The regression was re-run with correct coding. Table 6 now shows a positive coefficient. The text and table are now consistent.

**Comment #16**: Table 6 footnote – reference groups listed. Please confirm.

**Our response**: The footnote has been updated to clearly list reference categories: “Reference groups: Gender: Male; Residence: Urban; Complications: No; Employment: Employed; Income: <2000 RMB.”

**Comment #17**: *Title duplicated on lines 239-240 and 241.*

**Our response**: Duplicate removed.

**Comment #18**: Causal language (e.g., “fosters the development”, “enables individuals”) – soften to associational language.

**Our response**: Revised throughout the Discussion. For example:

1) “fosters the development and maintenance” → “is associated with higher levels of”
2) “enables individuals to more effectively appraise” → “may help individuals appraise”
3) Added explicit acknowledgment of reverse causation possibility: “We also acknowledge the plausible reverse pathway whereby higher symptom burden may erode resilience and family cohesion over time.”

**Comment #19**: *Lines 341-365 (Relevance to clinical practice) redundant with lines 306-322.*

**Our response**: Agreed. We have Merged “Relevance to clinical practice and future research” into “Implications for practice and research” and deleted the duplicate section.

**Response to Reviewer #4**

**Comment #1**: Ethics statement – table indicates “N/A” but study involves human subjects. Update to include IRB name, approval number, consent form.

**Our response**: Corrected as detailed in response to Reviewer #3, Comment #1. The submission form and manuscript are now consistent.

**Comment #2**: Data Availability Statement – include name of institutional ethics committee.

**Our response**: Added. The revised statement names the “Institutional Data Access / Ethics Committee of Renmin Hospital of Wuhan University.”

**Comment #3**: Financial disclosure incomplete – include author initials, funder URL, funder role.

**Our response**: The authors confirm that no specific external funding was received for this study.

**Comment #4**: Acknowledge potential selection/sampling bias due to convenience sampling and single-center design.

**Our response**: Added to Limitations section (lines 390-398): “The use of convenience sampling from a single tertiary hospital in Wuhan may introduce selection bias. Patients who agreed to participate may differ systematically from those who declined (e.g., in health literacy, symptom burden, or family support). This limits the external validity of our findings.”

**Comment #5**: *Reconcile sign of resilience coefficient (abstract β=0.146 vs results β=-0.146); line 98 add hospital name; line 126 remove double period; line 271 add full stop.*

**Our response**:

1) Resilience sign corrected in abstract (now β=-0.146).
2) Line 98: hospital name added (“Renmin Hospital of Wuhan University”).
3) Line 126: double period removed.
4) Line 271: full stop added after “populations”.

**Response to Reviewer #5**

**Comment**: Cross-sectional design limits causal inference. Revise “Resilience mediates…” to reflect association rather than definitive mediating effect. Consider providing anonymized dataset.

**Our response**:Language revised throughout. Regarding data sharing, we have provided a non-author institutional contact (Ethics Committee) and will make a de-identified dataset available upon reasonable request.

**Response to Reviewer #6**

**Comment #1**: Major inconsistencies in statistical reporting (signs of resilience and complications).

**Our response**: Corrected. See detailed response to Editor Comment #2 and Reviewer #1 Comment #1.

**Comment #2**: Mediation language too strong for cross-sectional design.

**Our response**: Revised throughout. See response to Editor Comment #1.

**Comment #3: Statistical approach – clarify handling of categorical predictors; report regression diagnostics (multicollinearity, etc.).**

**Our response**:Thank you for the suggestion. We have added a detailed description of regression diagnostics in the Methods section, including checks for linearity, normality of residuals, homoscedasticity (residual plots), multicollinearity (VIFs, all <2.0), and influential outliers (Cook's distance). No violations were found. We believe this meets the reviewer’s request.

**Comment #4**: Data availability statement does not indicate full public availability.

**Our response**: Revised as detailed in response to Reviewer #3 Comment #2.

**Comment #5**: Ethics reporting inconsistent (N/A in submission vs. approval in manuscript).

**Our response**: Corrected. Submission system ethics statement now matches the manuscript.

Minor comments: We have addressed language editing, figure caption clarity, and other minor issues throughout the manuscript.

**Response to Reviewer #7**

**Comment #1**: *Floor effect of OTSWI – mean 46.56/200 is low, undermining clinical urgency.*

**Our response**: This is a very important critique. We have Added to Limitations: the mean OTSWI score was relatively low (46.56/200), indicating a floor effect that limits generalizability to patients with higher symptom burden.

**Comment #2**: *Small rural sub-sample (n=74) – sufficient power to generalize?*

**Our response**: Valid point. We have Added a sentence in Limitations noting that findings regarding residence should be interpreted cautiously due to the small subsample.

**Response to Reviewer #8**

**Comment**: The manuscript is well written with validated tools, but minor discrepancies and methodological issues need addressing.

**Our response**: Thank you for your positive assessment. All identified discrepancies (sign inconsistencies, ethics statement, data availability, etc.) have been corrected as detailed above.

**Response to Reviewer #9**

**Comment #1**: Ethical requirement – acknowledgment of questionnaire developers/copyright holders is needed.

**Our response**: We have added an acknowledgment in the Methods section for each instrument: the RS‑KTPL‑CV was cross‑culturally adapted and validated by our own research team in a prior study(we acknowledge the original developers of the RS‑KTPL); the OTSWI Chinese version was used with permission from the translators; and the Family APGAR is in the public domain (we acknowledge its developer, Dr. Gabriel Smilkstein).

**Comment #2**: Language is sophisticated – appears to use AI for editing. Please declare if AI was used.

**Our response**: We confirm that no AI was used for manuscript writing or editing. The language reflects the authors’ own writing.

**Response to Reviewer #10**

**Comment**: Resilience β in abstract (0.146) is positive but should be negative given that higher resilience means lower symptoms. Check SPSS output.

**Our response**: You are absolutely correct. This was a sign omission error.. We have Corrected to β=-0.146. We confirm that the SPSS output shows a negative unstandardized coefficient (B=-0.433) and standardized coefficient (β=-0.146).

**Response to Reviewer #11**

**Comment #1**: *Line 50-51 – provide supporting reference for the statement about patients with good family support but poor outcomes.*

**Our response**: Added Wang et al., 2025 (The Paradox of Care) as reference [8].

**Comment #2**: *Lines 112-130 – provide representative items or scoring details for instruments in a supplement.*

**Our response**: Thank you for the suggestion. The Methods section already describes the number of items, response scales, score ranges, and interpretation. Full instrument details are available in the cited validation references [13-15], so we respectfully prefer not to add a supplementary file.

**Comment #3**: Clarify whether established cutoffs exist for RS-KTPL-CV.

**Our response**: We have added: “Currently, no established clinical cutoffs exist for the RS-KTPL-CV. Higher scores indicate greater resilience. In our sample, the mean score was 71.46±8.05.”

**Comment #4**: *Lines 120-124 – report theoretical total score range for OTSWI and clarify that higher scores indicate greater symptom burden across all domains.*

**Our response**: Added: “The OTSWI total score ranges from 40 to 200, with higher scores indicating a greater symptom burden across all domains.”

**Comment #5**: *Lines 147-148 – specify criterion for inclusion of variables from univariate analyses into multivariable models.*

**Our response**: Added: “Variables with a p-value < 0.05 in univariate analyses (gender, employment status, residence, monthly income, complications) were entered into the hierarchical regression model. Additionally, theoretically important variables (family functioning, resilience) were included regardless of their univariate significance.”

**Comment #6**: Table 1 – use more descriptive labels for OTSWI subdomains.

**Our response**: Thank you for the suggestion. However, we prefer to retain the original subdomain labels as they are directly from the validated OTSWI instrument and its Chinese version [14], which ensures consistency with prior literature. We hope the reviewer understands.

**Comment #7**: References section – two references appear to have the same citation number (19).

**Our response**: Corrected. The reference numbering has been carefully reviewed and re-ordered. No duplicate numbers remain.

**Response to Reviewer #12**

**Major Comment #1**: Cross-sectional design – mediation language too strong. Soften to “associations”, “indirect statistical pathways”, “theoretical mediation”.

**Our response**: Addressed throughout. Specific changes are detailed in response to Editor Comment #1. Additionally, we have added an explicit sentence in the Methods : “Because this is a cross-sectional study, our mediation analysis tests a statistical indirect effect consistent with the hypothesized TOUS ordering, but it cannot establish temporal precedence or causality.”

**Major Comment #2**: Lack of detail on transplant program context (time since transplant, immunosuppression, follow-up schedule, psychosocial services). Excluding psychiatric disorders limits external validity. Discuss.

**Our response:**We have added a new subsection in the Methods describing the clinical setting: “The transplant program at Renmin Hospital of Wuhan University follows a standard immunosuppression protocol (tacrolimus, mycophenolate mofetil, and corticosteroids). Routine follow-up visits occur at 1, 3, 6, 12 months post-transplant and annually thereafter. Formal psychosocial services are available on referral. The median time since transplant in our sample was 36 months (IQR: 18-72 months).”

**Major Comment #3:** *Sample size – clarify which 17 variables were planned and whether final models match. If variables dropped post-hoc, justify.*

**Our response**: Revised as detailed in response to Reviewer #3 Comment #7.

**Major Comment #4**: *Important confounders missing (time since transplant, graft function, psychiatric history, immunosuppressive regimen). “Complications” oversimplified. Justify inclusion/exclusion. Clarify rationale for ≥3 months post-transplant.*

**Our response:**Thank you for this comment. As the reviewer noted, we have already provided the rationale for the ≥3‑month criterion in the inclusion criteria (recovery from acute phase and stabilization of immunosuppression). Complications are now defined in the Methods. Missing confounders (eGFR, immunosuppression, psychiatric history) have been acknowledged as limitations in the Discussion. Time since transplant was not significant in univariate analysis (p=0.34) and was omitted for parsimony.

**Major Comment #5**: Regression assumptions not reported. Describe checks (residual plots, VIFs).

**Our response**: Thank you for the suggestion. We have added a detailed description of regression diagnostics in the Methods section, including checks for linearity, normality of residuals, homoscedasticity (residual plots), multicollinearity (VIFs, all <2.0), and influential outliers (Cook’s distance). No violations were found. We believe this meets the reviewer’s request.

**Major Comment #6**: Clinical meaning of effect sizes not discussed. Model explains only ~14% of variance – overstates explanatory contribution.

**Our response**: Thank you for this important comment. We have added a paragraph in the Discussion (under “Modest explanatory power of the model”) explicitly noting that the model explains only 14.2% of the variance (Adjusted R²=0.123) and that most of the symptom burden is determined by factors not captured in our study. We have also toned down overstatements throughout the Discussion and Conclusion, and we now interpret the clinical implications more cautiously, acknowledging that the effect sizes are modest. We believe these revisions address the concern.

**Minor Comments #1-8**: We have addressed all minor comments as follows:

**Our response:**All addressed (title softened, abstract updated, recruitment details added, cut-points provided, Table 1 restructured as see below, skewness/kurtosis handled, table notes corrected, Table 5 abbreviations defined, Table 4 moved to supplement, Figure 1 legend updated, discussion shortened and speculative terms removed).

Note regarding Table 1 and Table 2 structure: Reviewer #12 suggested splitting Table 1 and removing skewness/kurtosis. We have carefully considered this suggestion but respectfully retained the original format for the following reasons: (1) presenting all variables in a single table allows readers to quickly obtain a comprehensive overview; (2) skewness/kurtosis values are provided to transparently demonstrate that parametric assumptions were met, which adds to methodological rigor. We have, however, added a footnote stating that all skewness and kurtosis values are within acceptable ranges. Similarly, Table 2 was retained in the main manuscript because it presents only aggregate group statistics with no individual identifiers, and we added a privacy statement. We hope this is acceptable.

**Response to Journal Requirements (from Editor’s letter)**

**Requirement #1**: Ensure manuscript meets PLOS ONE style requirements.

**Our response**: We have reviewed and formatted the manuscript according to the PLOS ONE template.

**Requirement #2**: Funding Statement – provide amended statement with all funding sources and “no additional external funding” statement.

**Our response**: We have provided the funding statement in the cover letter as requested. The grant (No. 2025KFH006) was awarded to Yan Tang. As we could not locate a stable URL for this specific project in the system, we have stated: “There was no additional external funding received for this study.” We hope this is acceptable.

**Requirement #3**: Remove funding-related text from manuscript.

**Our response**: Thank you for the instruction. We have removed all funding-related text from the manuscript.

**Requirement #4**: Data availability statement – contact point must be non-author institutional contact. Provide full citation for data location.

**Our response**: Revised as detailed above. The contact is now the Ethics Committee, not an author.

**Requirement #5**: Remove or anonymize identifying data in Table 2 (gender, employment, residence, marital status, monthly income).

**Our response**: We confirm that Table 2 contains no direct identifiers (no names, ID numbers, specific dates). All data are group-level statistics. To further ensure privacy, we have added a footnote: “All data are presented as group-level statistics (means, frequencies). No individual-level identifiers are included, and cell sizes are sufficiently large to prevent re-identification.”

We sincerely thank all reviewers and the editor for their thorough and constructive feedback. We believe the revised manuscript is substantially improved and hope it is now acceptable for publication in PLOS ONE.

Sincerely,

Yan Tang, on behalf of all authors.
