# Supplementary material for: Resilience and its mediating role in the association between family functioning and symptom experience in kidney transplant recipients: A cross-sectional study
Source: PLoS One. 2026 Nov 12;21(11):e0361968. doi: 10.1371/journal.pone.0361968 (PMC13759052; doi:10.1371/journal.pone.0361968)
Supplement: Supplementary file 6 [file pone.0361968.s008.docx]

**Response to Reviewers**

Manuscript ID: PONE-D-26-00026R1

Title: Resilience and its mediating role in the association between family functioning and symptom experience in kidney transplant recipients: A cross-sectional study

Dear Dr. Kaewput and Reviewers,

Thank you for your further evaluation of our revised manuscript. We are grateful for the remaining constructive comments, which have helped us further improve the paper. Below we provide point‑by‑point responses to all new comments raised in this round. **All changes are highlighted in the revised manuscript using Track Changes.**

**Response to Reviewer #4**

**Comment #1** (Ethics Statement harmonization): Please ensure that the Ethics Statement is fully harmonized across the manuscript and the submission information, particularly between the ethics table and the "Ethical Approval" section where there is "N/A" in the table yet a description under the ethical approval.

**Our response:** We confirm that the “N/A” entry in the submission system refers only to the absence of a specified expiration date in the ethics approval document issued by the Medical Ethics Committee of Renmin Hospital of Wuhan University. This is consistent with the committee’s standard practice for non‑interventional studies (cross‑sectional questionnaire surveys), where approval is granted without a fixed expiry date, provided the study is conducted in accordance with the approved protocol. The ethics approval itself (Approval No. WDRY2024‑K282) and the informed consent procedures are fully described in the manuscript (“Ethical approval” section).

**Comment #2** (Language proofreading): I recommend one final round of proofreading to correct minor formatting and grammatical inconsistencies, particularly around citations, spacing, and punctuation. For example: Line 311 missing full stop; Line 369 missing article; Line 370 word form.

**Our response**: We thank the reviewer for the careful reading. We have corrected all the identified issues: (1) added a full stop after “populations” in the discussion of gender differences; (2) changed “such dual-path approach” to “such a dual-path approach”; (3) changed “potential mitigating” to “potentially mitigating.” We have also performed a final proofread of the entire manuscript to address other minor formatting and grammatical inconsistencies.

**Response to Reviewer #9**

**Comment #1** (Questionnaire acknowledgment): In order to give full credit, ideally you should give a link to from where you downloaded the questionnaire and under acknowledgment write sentences thanking the original developers of the study. Even though you used modified version, you still need to acknowledge the original developer.

**Our response**: We agree with the reviewer and have added formal acknowledgment of the original developers of all three instruments (RS-KTPL, OTSWI, and Family APGAR) in the Acknowledgments section at the end of the manuscript. We also thank the translators of the Chinese version of the OTSWI in the same section.

**Comment #2** (Permission letter): You need to attach in the appendix the permission letter that you obtained from one of the developers.

**Our response**: We do not have a formal written permission letter to attach. We confirm the following: (1) The Family APGAR is a public‑domain instrument and does not require permission; (2) The RS‑KTPL‑CV was cross‑culturally adapted and validated by our own research team [13], and we acknowledge the original developers in the Acknowledgments; (3) The Chinese version of the OTSWI was obtained from the translators for academic research purposes and was used in accordance with its intended use, with appropriate citation provided [14]. As there was no formal written permission letter, we are unable to include one in the supplementary materials. We have, however, added clear acknowledgments of the original developers in the manuscript to give full credit.

**Comment #3** (Reference format – Vancouver style, remove PMCID): Your references are not in accordance with the Vancouver style. They do not need PMCID numbers at the end.

**Our response**: We have revised all references according to PLOS ONE’s Vancouver style requirements. All PubMed PMID and PMCID numbers have been removed, and only essential citation information (authors, title, journal, year, volume, pages, DOI) is retained.

**Response to Reviewer #11**

**Comment #1** (Grammar in Methods scoring description, Lines 141-142): The description of the scoring contains a grammatical inconsistency.

**Our response:** We have revised the scoring description in the Methods section from “Higher total scores indicating a greater symptom burden” to “Higher total scores indicate a greater symptom burden” to correct the grammatical inconsistency.

**Comment #2** (OTSWI subdomain labels in Table 1): The labels currently used in Table 1 do not in fact correspond to the original OTSWI subdomain labels... several of the abbreviated labels are ambiguous to readers.

**Our response**: We thank the reviewer for this careful observation. We acknowledge that the previously used labels were descriptive summaries rather than the original factor labels. To address this, we have revised the Table 1 subdomain labels to align with the seven‑factor structure of the Chinese version of the OTSWI as reported by Shi et al. The revised labels are: Sleeping problems, Joint and muscle pain, Fatigue, Cognitive functioning, Basic ADL, Mood, and Economy.

**Additional Note**

During this revision, the affiliation of the first and corresponding author (Dr. Yan Tang) has been updated to reflect her current institution: Hemodialysis Center, The Sixth Hospital of Wuhan, Affiliated Hospital of Jianghan University, Wuhan, China. All other authors’ affiliations remain unchanged. This change has been made in both the manuscript and the submission system.We hope that the revised manuscript is now acceptable for publication in PLOS ONE. Thank you again for your time and consideration.

Sincerely,

Yan Tang, on behalf of all authors

Hemodialysis Center, The Sixth Hospital of Wuhan, Affiliated Hospital of Jianghan University, Wuhan, China

E‑mail: 1348580254@qq.com
