# Supplementary material for: Resilience and its mediating role in the association between family functioning and symptom experience in kidney transplant recipients: A cross-sectional study
Source: PLoS One. 2026 Nov 12;21(11):e0361968. doi: 10.1371/journal.pone.0361968 (PMC13759052; doi:10.1371/journal.pone.0361968)
Supplement: Supplementary file 7 [file pone.0361968.s009.docx]

# **Response to Reviewers**

**Manuscript ID:** PONE-D-26-00026R2
**Title:** Resilience and its mediating role in the association between family functioning and symptom experience in kidney transplant recipients: A cross-sectional study

**Dear Dr. Kaewput and Reviewers,**

Thank you for the further evaluation of our revised manuscript. We have carefully addressed the remaining comments. Below we provide a point-by-point response.

## **Response to Academic Editor**

**Comment #1:** Please explicitly confirm whether there is any participant overlap between the present cohort of 333 kidney transplant recipients and the cohort reported in Tang et al. (2025).

**Our response:** We confirm that the two datasets were collected at different time periods. Because both studies recruited kidney transplant recipients from the same outpatient clinic, we cannot exclude the possibility of partial participant overlap. However, the exact extent of overlap cannot be precisely determined retrospectively, as the two studies used separate data collection rounds and the prior validation study did not record a shared unique identifier. Importantly, the two studies address entirely different research questions: Tang et al. (2025) focused exclusively on the cross-cultural adaptation and psychometric validation of the RS-KTPL instrument, whereas the present study examines the mediating role of resilience in the association between family functioning and symptom experience. The analytical approaches, hypotheses, and outcome variables are distinct. All data were collected under the same ethics approval (WDRY2024-K282). We believe the two papers do not duplicate findings and that the present study represents an independent research contribution.

**Comment #2:** Please recheck the statistical values reported in Tables 2 and 5 against the original output.

**Our response:** We have rechecked all statistical values in Tables 2 and 5 against the original SPSS output. In Table 2, the t statistics for gender, residence, and complications were corrected to **t = 2.49, t = 2.80, and t = 4.00**, respectively (p values unchanged). In Table 5, the negative t value for complications was a transcription error and has been corrected to a positive value (Model 1: **t = 3.700**; Model 2: **t = 3.242**), consistent with the positive regression coefficient. We confirm that all statistical conclusions remain unchanged.

**Comment #3:** Please clarify how employment status and monthly income were coded in the regression analyses. As these are multi-category variables, particularly employment status, appropriate indicator/dummy coding should be used.

**Our response:** We acknowledge the editor’s concern. We have re-run the hierarchical regression using dummy coding for both variables. Specifically:

**Employment status** was coded with **“Other”** as the reference category, creating two dummy variables: “Employed vs Other” and “Retired vs Other”.

**Monthly income** was coded with **“5000–10000”** as the reference category, creating three dummy variables: “<2000 vs 5000–10000”, “2000–5000 vs 5000–10000”, and “>10000 vs 5000–10000”.

The updated results are presented in the revised **Table 4** (model summary) and **Table 5** (regression coefficients). The coding scheme is provided in **Supplementary Table S2**. Importantly, the main findings remain unchanged: resilience (**β = −0.152, p = .005**), family functioning (**β = −0.124, p = .022**), and complications (**β = 0.169, p = .001**) remained significant predictors of symptom experience in the final model. We believe this fully addresses the editor’s concern.

## **Response to Reviewer #9**

**Comment:** Good amendments overall. However, before publishing please confirm the copyright rules and regulations as you do not have the permission letter.

**Our response:** We thank the reviewer for this important reminder. We confirm the following regarding the copyright status of each instrument:

**Family APGAR:** This instrument is in the public domain and freely available for research use. No permission letter is required. We acknowledge Dr. Gabriel Smilkstein as the original developer in the Acknowledgments section.

**RS-KTPL-CV:** This scale was cross-culturally adapted and validated by our own research team in a prior study [13]. We acknowledge the original developers of the RS-KTPL in the Acknowledgments section. No external permission is required for our own adapted version.

**OTSWI (Chinese version):** The Chinese version was obtained from the translators for academic research purposes. It is not a copyrighted commercial instrument, and no formal written permission letter was required. We have appropriately cited the original validation study [14] and acknowledged both the original developers and the Chinese translators in the Acknowledgments section.

## **Response to Reviewer #11**

**Comment #1:** The scoring metric described in the Methods differs from that of the Chinese version cited as the source [14]… Table 1 values do not appear compatible with 1-to-5 scoring… Please confirm which metric was used and reconcile the Methods, Table 1, and the derived statement in the Discussion (“46.56/200”).

**Our response:** We thank the reviewer for this very careful observation. We have rechecked the scoring of the Chinese version of the OTSWI. The instrument uses a **0–4 Likert scale (0 = never, 4 = always)**, with a total score range of **0–160**. We have corrected the Methods section accordingly:

“Items are rated on a 5-point Likert scale from 0 (never) to 4 (always), with total scores ranging from 0 to 160.”

We have also corrected the reference to the total score in the Discussion (“**46.56/160**” instead of “46.56/200”), and the characterization of symptom burden has been harmonized to “**relatively low**” throughout the manuscript. We apologize for these inconsistencies.

**Comment #2:** “Joint and muscle pain” is a descriptive summary rather than the label used in the source instrument (“Ache of lower limb”). Please correct the name of this label.

**Our response:** Corrected. The label in Table 1 has been changed to **“Ache of lower limb”** to align with the original OTSWI instrument. We have also added a footnote to Table 1 clarifying that the subdomain labels are based on the original OTSWI factor structure.

We hope the revised manuscript is now suitable for publication in PLOS ONE. Thank you again for your time and consideration.

Sincerely,

**Yan Tang, on behalf of all authors**
Hemodialysis Center, The Sixth Hospital of Wuhan, Affiliated Hospital of Jianghan University, Wuhan, China
E-mail: 1348580254@qq.com
