# Supplementary material for: Resilience and its mediating role in the association between family functioning and symptom experience in kidney transplant recipients: A cross-sectional study
Source: PLoS One. 2026 Nov 12;21(11):e0361968. doi: 10.1371/journal.pone.0361968 (PMC13759052; doi:10.1371/journal.pone.0361968)
Supplement: Supplementary file 8 [file pone.0361968.s010.docx]

**Dear Dr. Kaewput,**

Thank you for your careful review and for identifying the remaining inconsistencies. We have now addressed all four issues in full. Below we reproduce each of your comments in its entirety, followed by our point-by-point response. The revised manuscript with tracked changes and a clean version have been uploaded.

**Comment 1:** *The regression coefficients and p values for resilience and family functioning reported in the response letter do not appear to match those reported in the revised manuscript. Please verify the final values against the original SPSS output and ensure that the Abstract, Results, Tables, and response letter are fully consistent.*

**Our response:** We have rechecked all reported values against the original SPSS output. The following inconsistencies were identified and corrected:

- In the **Abstract**, the values were already correct: resilience (β = −0.152, p = .005), family functioning (β = −0.124, p = .022).
- In the **Results text** (paragraph beginning “The final regression model (Table 5) showed…”), the values had not been updated and still read: complications (β = 0.176, p = .001), resilience (β = −0.146, p = .007), family functioning (β = −0.123, p = .023). These have now been corrected to: complications (β = **0.169**, p = .001), resilience (β = **−0.152**, p = .005), family functioning (β = **−0.124**, p = .022).
- In **Table 5**, the coefficients for resilience and family functioning were already updated in the previous round. We have now verified that all values in the table match the SPSS output: resilience (B = −0.452, SE = 0.158, β = −0.152, t = −2.854, p = .005), family functioning (B = −1.097, SE = 0.476, β = −0.124, t = −2.305, p = .022).
- The **response letter** has been updated accordingly to reflect these final values.

The Abstract, Results, Tables, and this response letter are now fully consistent.

**Comment 2:** *The text states that residence was included among the demographic and clinical variables entered into Model 1, whereas residence does not appear in the Table 4 footnote or Table 5. Please clarify whether residence was included in the final regression model and revise the Methods, Results, and tables accordingly.*

**Our response:** We confirm that residence was included as a demographic covariate in both Model 1 and Model 2. However, it had been omitted from the Table 4 footnote and from Table 5. We have made the following corrections:

- **Table 4 footnote** now reads: “Model 1 predictors: gender, employment status (dummy variables), **residence**, monthly income (dummy variables), and complications. Model 2 predictors: Model 1 variables plus resilience and family functioning.”
- **Table 5** now includes the residence row for both models:
  - Model 1: Residence (Rural vs Urban): B = 6.186, SE = 3.240, β = 0.108, t = 1.909, p = .057, 95% CI [−0.19, 12.56].
  - Model 2: Residence (Rural vs Urban): B = 5.950, SE = 3.178, β = 0.104, t = 1.872, p = .062, 95% CI [−0.30, 12.20].
- The **Methods** section already stated that residence was among the variables entered into Model 1, so no change was needed there.

**Comment 3:** *In Table 5, “Employment: Other vs Other” appears to be a labeling error and should presumably read “Employed vs Other.” Please verify and correct this.*

**Our response:** Corrected. The row previously labeled “Employment: Other vs Other” has been changed to **“Employment: Employed vs Other”**. The reference category remains “Other” for employment status. All other labels in Table 5 have been checked and are correct.

**Comment 4:** *Please ensure that the description of the relationship between the present cohort and the previous Tang et al. (2025) study accurately reflects your response that partial participant overlap cannot be excluded.*

**Our response:** We have added a clear statement in the **Limitations** section of the manuscript. The added text reads:

“The present cohort partially overlaps with the sample reported in our prior methodological study (Tang et al., 2025), which focused on the validation of the RS-KTPL instrument. Because the two studies recruited from the same outpatient clinic at different time periods, partial participant overlap cannot be excluded. However, the two studies address different research questions and report different outcomes.”

This statement accurately reflects our previous response that partial overlap cannot be excluded. It has been placed at the end of the Limitations paragraph (after the fifth limitation).

The manuscript is now internally consistent and ready for acceptance. Thank you again for your time and consideration.

Sincerely,

**Yan Tang, on behalf of all authors**
Hemodialysis Center, The Sixth Hospital of Wuhan, Affiliated Hospital of Jianghan University
E-mail: 1348580254@qq.com
