# Supplementary material for: Resilience and its mediating role in the association between family functioning and symptom experience in kidney transplant recipients: A cross-sectional study
Source: PLoS One. 2026 Nov 12;21(11):e0361968. doi: 10.1371/journal.pone.0361968 (PMC13759052; doi:10.1371/journal.pone.0361968)
Supplement: Supplementary file 9 [file pone.0361968.s001.doc]

STROBE Statement—Checklist of items that should be included in reports of ***cross-sectional studies***

|  | Item No | Recommendation | In the manuscript |
| --- | --- | --- | --- |
| **Title and abstract** | 1 | (*a*) Indicate the study’s design with a commonly used term in the title or the abstract | Title: “Resilience and its mediating role … a cross-sectional study”. Abstract: line 1. |
|  |  | (*b*) Provide in the abstract an informative and balanced summary of what was done and what was found | Abstract, lines 1–32 (Objective, Methods, Results, Conclusion). |
| Introduction | | |  |
| Background/rationale | 2 | Explain the scientific background and rationale for the investigation being reported | Introduction, lines 1–69 (from “Kidney transplantation is the treatment…” to the end of the TOUS framework). |
| Objectives | 3 | State specific objectives, including any prespecified hypotheses | Introduction, “Aims and Hypotheses”, lines 70–77. |
| Methods | | |  |
| Study design | 4 | Present key elements of study design early in the paper | “Study design”, lines 80–82: “A cross-sectional, questionnaire-based study was conducted and reported following STROBE guidelines.” |
| Setting | 5 | Describe the setting, locations, and relevant dates, including periods of recruitment, exposure, follow-up, and data collection | “Participants and settings”, lines 84–86: “recruited between January and February 2025 from the outpatient follow-up clinic of a tertiary Grade A hospital in Wuhan, China.” Also lines 100–102 (data collection dates). |
| Participants | 6 | (*a*) Give the eligibility criteria, and the sources and methods of selection of participants | “Participants and settings”, lines 87–95: inclusion and exclusion criteria, plus definition of comorbidities and stressful life events. |
| Variables | 7 | Clearly define all outcomes, exposures, predictors, potential confounders, and effect modifiers. Give diagnostic criteria, if applicable | Outcome: “Symptom experience” (OTSWI, lines 120–124). Exposure/Predictor: “Family functioning” (Family APGAR, lines 127–130). Mediator: “Resilience” (RS-KTPL-CV, lines 116–119). Confounders: gender, complications, etc. (Table 5, footnote). |
| Data sources/ measurement | 8* | For each variable of interest, give sources of data and details of methods of assessment (measurement). Describe comparability of assessment methods if there is more than one group | *“Measures” subsections, lines 112–130: each instrument described with number of items, response scale, score range, and reliability (Cronbach’s α).* |
| Bias | 9 | Describe any efforts to address potential sources of bias | “Data collection” (forced‑response, attention check, lines 101–109); “Data analysis” (anonymity, reverse‑scored items, line 133); Harman’s single‑factor test (lines 217–222). |
| Study size | 10 | Explain how the study size was arrived at | “Participants and settings”, lines 96–104: sample size calculation based on 5‑10 events per variable, accounting for 20% invalid response rate. |
| Quantitative variables | 11 | Explain how quantitative variables were handled in the analyses. If applicable, describe which groupings were chosen and why | “Data analysis”, lines 144–152 (hierarchical regression, variable selection). For employment and income treated as ordinal, see “Modest explanatory power” and limitations (lines 274–285) plus Supplementary Table S2. |
| Statistical methods | 12 | (*a*) Describe all statistical methods, including those used to control for confounding | “Data analysis”, lines 131–161: descriptive statistics, t‑tests/ANOVA, Pearson correlation, hierarchical regression, PROCESS mediation (Model 4), bootstrap. Confounders: gender and complications included as covariates (line 161). |
|  |  | (*b*) Describe any methods used to examine subgroups and interactions | Not performed. No subgroup or interaction analyses were planned. |
|  |  | (*c*) Explain how missing data were addressed | No missing data: electronic questionnaire with forced‑response settings (line 103). |
|  |  | (*d*) If applicable, describe analytical methods taking account of sampling strategy | Single‑center convenience sample; no weighting or complex sampling methods used. Acknowledged in limitations (lines 277–280). |
|  |  | (*e*) Describe any sensitivity analyses | No formal sensitivity analyses performed. |
| Results | | |  |
| Participants | 13* | (a) Report numbers of individuals at each stage of study—eg numbers potentially eligible, examined for eligibility, confirmed eligible, included in the study, completing follow-up, and analysed | “Data collection”, lines 101–109: 335 approached, 2 invalid, 333 final. No flow diagram but numbers reported. |
|  |  | (b) Give reasons for non-participation at each stage | Refusal rate not recorded; two invalid responses due to failed attention check and straight‑line pattern (line 108). Acknowledged as limitation. |
|  |  | (c) Consider use of a flow diagram | Not used; numbers are small and clearly described in text. |
| Descriptive data | 14* | (a) Give characteristics of study participants (eg demographic, clinical, social) and information on exposures and potential confounders | Table 2 (lines 192‑194) and text lines 173‑178. Key characteristics: gender, employment, residence, income, complications. |
|  |  | (b) Indicate number of participants with missing data for each variable of interest | No missing data (forced‑response questionnaire). |
| Outcome data | 15* | Report numbers of outcome events or summary measures | Outcome (OTSWI total score) mean and SD: 46.56±23.86 (Table 1, line 179). Subdomain scores also presented. |
| Main results | 16 | (*a*) Give unadjusted estimates and, if applicable, confounder-adjusted estimates and their precision (eg, 95% confidence interval). Make clear which confounders were adjusted for and why they were included | Unadjusted: Table 2 (group comparisons) and Table 3 (correlations). Adjusted: Table 5 (hierarchical regression) with 95% CIs. Confounders: gender, employment, residence, income, complications (selected based on univariate significance and theory, line 144‑148). |
|  |  | (*b*) Report category boundaries when continuous variables were categorized | ncome categories (¥ <2000, 2000‑5000, 5000‑10000, >10000) and employment categories (employed, retired, other) are reported in Table 2 footnote and Supplementary Table S2. |
|  |  | (*c*) If relevant, consider translating estimates of relative risk into absolute risk for a meaningful time period | Not applicable (no relative risk). |
| Other analyses | 17 | Report other analyses done—eg analyses of subgroups and interactions, and sensitivity analyses | Mediation analysis (PROCESS) reported in Table 6 and Figure 1. No subgroup or interaction analyses. |
| Discussion | | |  |
| Key results | 18 | Summarise key results with reference to study objectives | “Summary of key findings”, lines 238‑246. |
| Limitations | 19 | Discuss limitations of the study, taking into account sources of potential bias or imprecision. Discuss both direction and magnitude of any potential bias | “Limitations and future directions”, lines 274‑285: cross‑sectional design, convenience sample, floor effect, self‑report bias, missing confounders, exclusion of psychiatric disorders. |
| Interpretation | 20 | Give a cautious overall interpretation of results considering objectives, limitations, multiplicity of analyses, results from similar studies, and other relevant evidence | “Interpretation of findings within the context of the literature” (lines 248‑272) and “Conclusion” (lines 287‑291). |
| Generalisability | 21 | Discuss the generalisability (external validity) of the study results | “Limitations and future directions”, lines 277‑280 (single centre, rural subsample, medically stable patients only). |
| Other information | | |  |
| Funding | 22 | Give the source of funding and the role of the funders for the present study and, if applicable, for the original study on which the present article is based | No funding received. |
|  |  |  |  |
|  |  |  |  |
|  |  |  |  |
|  |  |  |  |
|  |  |  |  |
|  |  |  |  |
|  |  |  |  |
|  |  |  |  |
|  |  |  |  |
|  |  |  |  |
|  |  |  |  |
|  |  |  |  |
|  |  |  |  |
|  |  |  |  |
|  |  |  |  |
|  |  |  |  |
|  |  |  |  |
|  |  |  |  |
|  |  |  |  |
|  |  |  |  |
|  |  |  |  |
|  |  |  |  |
|  |  |  |  |
|  |  |  |  |
|  |  |  |  |
|  |  |  |  |
|  |  |  |  |
|  |  |  |  |
|  |  |  |  |
|  |  |  |  |
|  |  |  |  |
|  |  |  |  |
|  |  |  |  |
|  |  |  |  |
|  |  |  |  |
|  |  |  |  |
|  |  |  |  |
|  |  |  |  |

*Give information separately for exposed and unexposed groups.

**Note:** An Explanation and Elaboration article discusses each checklist item and gives methodological background and published examples of transparent reporting. The STROBE checklist is best used in conjunction with this article (freely available on the Web sites of PLoS Medicine at http://www.plosmedicine.org/, Annals of Internal Medicine at http://www.annals.org/, and Epidemiology at http://www.epidem.com/). Information on the STROBE Initiative is available at www.strobe-statement.org.
