# Supplementary material for: Resilience and its mediating role in the association between family functioning and symptom experience in kidney transplant recipients: A cross-sectional study
Source: PLoS One. 2026 Nov 12;21(11):e0361968. doi: 10.1371/journal.pone.0361968 (PMC13759052; doi:10.1371/journal.pone.0361968)
Supplement: Peer review history

# Peer review: Decision Letter 0

**DOI:** 10.1371/journal.pone.0361968.r001

**Authors:** Wisit Kaewput

14 May 2026

Dear Dr. TANG,

Thank you for submitting your manuscript to PLOS ONE. After careful consideration, we feel that it has merit but does not fully meet PLOS ONE’s publication criteria as it currently stands. Therefore, we invite you to submit a revised version of the manuscript that addresses the points raised during the review process.

Please submit your revised manuscript by Jun 28 2026 11:59PM. If you will need more time than this to complete your revisions, please reply to this message or contact the journal office at plosone@plos.org. When you're ready to submit your revision, log on to https://www.editorialmanager.com/pone/ and select the 'Submissions Needing Revision' folder to locate your manuscript file.

- A letter that responds to each point raised by the academic editor and reviewer(s). You should upload this letter as a separate file labeled 'Response to Reviewers'.
- A marked-up copy of your manuscript that highlights changes made to the original version. You should upload this as a separate file labeled 'Revised Manuscript with Track Changes'.
- An unmarked version of your revised paper without tracked changes. You should upload this as a separate file labeled 'Manuscript'.

If you would like to make changes to your financial disclosure, please include your updated statement in your cover letter. Guidelines for resubmitting your figure files are available below the reviewer comments at the end of this letter.

If applicable, we recommend that you deposit your laboratory protocols in protocols.io to enhance the reproducibility of your results. Protocols.io assigns your protocol its own identifier (DOI) so that it can be cited independently in the future. For instructions see: https://journals.plos.org/plosone/s/submission-guidelines#loc-laboratory-protocols. Additionally, PLOS ONE offers an option for publishing peer-reviewed Lab Protocol articles, which describe protocols hosted on protocols.io. Read more information on sharing protocols at https://plos.org/protocols?utm_medium=editorial-email&utm_source=authorletters&utm_campaign=protocols.

As the corresponding author, your ORCID iD is verified in the submission system and will appear in the published article. PLOS supports the use of ORCID, and we encourage all coauthors to register for an ORCID iD and use it as well. Please encourage your coauthors to verify their ORCID iD within the submission system before final acceptance, as unverified ORCID iDs will not appear in the published article. *Only*  the individual author can complete the verification step; PLOS staff *cannot*  verify ORCID iDs on behalf of authors.

We look forward to receiving your revised manuscript.

Kind regards,

Wisit Kaewput, MD, FRCP(London), FASN, FAcadMEd

Academic Editor

PLOS One

Journal Requirements:

When submitting your revision, we need you to address these additional requirements.

1. Please ensure that your manuscript meets PLOS ONE's style requirements, including those for file naming. The PLOS ONE style templates can be found at https://journals.plos.org/plosone/s/file?id=wjVg/PLOSOne_formatting_sample_main_body.pdf and https://journals.plos.org/plosone/s/file?id=ba62/PLOSOne_formatting_sample_title_authors_affiliations.pdf

2. Thank you for stating in your Funding Statement:

[This work was supported by the Hubei Provincial Key Laboratory Open Project - Nursing Special Project (Grant No. 2025KFH006).].

Please provide an amended statement that declares *all* the funding or sources of support (whether external or internal to your organization) received during this study, as detailed online in our guide for authors at http://journals.plos.org/plosone/s/submit-now.  Please also include the statement “There was no additional external funding received for this study.” in your updated Funding Statement.

Please include your amended Funding Statement within your cover letter. We will change the online submission form on your behalf.

3. Please remove any funding-related text from the manuscript. Please note that funding information should not appear in any section or other areas of your manuscript. We will publish the funding statement that is present in the Financial Disclosure section of the online submission form.

4. In the online submission form you indicate that your data is not available for proprietary reasons and have provided a contact point for accessing this data. Please note that your current contact point is a co-author on this manuscript. According to our Data Policy, the contact point must not be an author on the manuscript and must be an institutional contact, ideally not an individual. Please revise your data statement to a non-author institutional point of contact, such as a data access or ethics committee, and send this to us via return email. Please also include contact information for the third-party organization, and please include the full citation of where the data can be found.

5. Please include a copy of Table 6 which you refer to in your text on page 10.

6. We note that there is identifying data in Table 2. Prior to sharing human research participant data, authors should consult with an ethics committee to ensure data are shared in accordance with participant consent and all applicable local laws.

Data sharing should never compromise participant privacy. It is therefore not appropriate to publicly share personally identifiable data on human research participants. The following are examples of data that should not be shared:

-Name, initials, physical address

-Ages more specific than whole numbers

-Internet protocol (IP) address

-Specific dates (birth dates, death dates, examination dates, etc.)

-Contact information such as phone number or email address

-Location data

-ID numbers that seem specific (long numbers, include initials, titled “Hospital ID”) rather than random (small numbers in numerical order)

Data that are not directly identifying may also be inappropriate to share, as in combination they can become identifying. For example, data collected from a small group of participants, vulnerable populations, or private groups should not be shared if they involve indirect identifiers (such as sex, ethnicity, location, etc.) that may risk the identification of study participants.

Additional guidance on preparing raw data for publication can be found in our Data Policy (https://journals.plos.org/plosone/s/data-availability#loc-human-research-participant-data-and-other-sensitive-data) and in the following article: http://www.bmj.com/content/340/bmj.c181.long.

Please remove or anonymize all personal information (gender, employment status, residence, marital status, monthly income), ensure that the data shared are in accordance with participant consent, and add an anonymized version to your manuscript file.

If the reviewer comments include a recommendation to cite specific previously published works, please review and evaluate these publications to determine whether they are relevant and should be cited. There is no requirement to cite these works unless the editor has indicated otherwise.

Additional Editor Comments:

This manuscript examines the relationships among family functioning, resilience, and symptom experience in kidney transplant recipients, with a proposed mediation model in which resilience partially mediates the association between family functioning and symptom burden. The topic is clinically relevant, particularly because psychosocial factors are increasingly recognised as important determinants of post-transplant well-being. The use of established instruments, including the Family APGAR Index and the Organ Transplant Symptom and Well-being Inventory, is a strength, and the authors have attempted to frame the work using the Theory of Unpleasant Symptoms. The study also reports ethical approval, clear inclusion and exclusion criteria, and a reasonable sample size of 333 participants.

However, I recommend major revision before the manuscript can be considered further. My main concern is that the central conclusion should be stated more cautiously in view of the cross-sectional design. The authors present resilience as having a “mediating role” between family functioning and symptom experience, but the data were collected at a single time point. Although statistical mediation can be estimated in cross-sectional data, this design cannot establish temporal ordering or causality. It is also plausible that greater symptom burden may reduce perceived resilience and family functioning, or that unmeasured psychological, socioeconomic, or clinical factors may influence all three constructs. The authors acknowledge this limitation, but the abstract, discussion, conclusion, and clinical implications should be revised to avoid language that implies a confirmed causal or mechanistic pathway.

There are also several statistical and reporting issues that require clarification. The regression results appear internally inconsistent. For example, the manuscript states that complications were the strongest positive predictor of higher symptom burden, yet the coefficient shown for complications is negative in both Model 1 and Model 2, despite the coding indicating “No = 0, Yes = 1.” This may reflect a coding issue, a table-labeling error, or an interpretation problem. Similarly, the abstract reports resilience as β = 0.146, although the expected and subsequently reported association with symptom burden is negative. These discrepancies should be corrected and the direction of all coefficients carefully checked throughout the manuscript.

The explanatory value of the model should also be interpreted more conservatively. The full model explains 14.2% of the variance in symptom experience, with psychosocial variables adding approximately 3.9% beyond demographic and clinical covariates. While this finding may still be clinically meaningful, the discussion should avoid overstating the practical implications. Recommendations for systematic screening, family-centred consultation, resilience training, and integrated care pathways should be framed as potential implications or hypotheses for future testing rather than as direct conclusions from the current observational study.

The sampling strategy also needs fuller discussion. Participants were recruited by convenience sampling from a single tertiary Grade A hospital in Wuhan over a short two-month period. This may limit generalisability and introduce selection bias, particularly because participants had to be medically stable, able to complete an electronic questionnaire, and free from major comorbidities or stressful life events during the study period. These criteria may have excluded more vulnerable transplant recipients, who may experience higher symptom burden. The authors should discuss how this may affect the applicability of the findings to broader transplant populations.

Finally, the analytic approach should be reported more clearly. The covariates included in the mediation model are described only as “significant covariates,” with examples such as gender and complications, but the exact model specification should be provided. The handling of ordinal variables such as income and employment status also requires justification. In addition, Harman’s single-factor test alone is not a strong basis for excluding common method bias in a study where all major variables were self-reported at the same time point. The authors should either provide additional justification or acknowledge this limitation more explicitly.

[Note: HTML markup is below. Please do not edit.]

Reviewers' comments:

Reviewer's Responses to Questions

**Comments to the Author**

1. Is the manuscript technically sound, and do the data support the conclusions?

Reviewer #1: Partly

Reviewer #2: Yes

Reviewer #3: Partly

Reviewer #4: Yes

Reviewer #5: Partly

Reviewer #6: Partly

Reviewer #7: Yes

Reviewer #8: Yes

Reviewer #9: Yes

Reviewer #10: Yes

Reviewer #11: Yes

Reviewer #12: Partly

**********

2. Has the statistical analysis been performed appropriately and rigorously?

Reviewer #1: Yes

Reviewer #2: Yes

Reviewer #3: No

Reviewer #4: Yes

Reviewer #5: Yes

Reviewer #6: No

Reviewer #7: Yes

Reviewer #8: Yes

Reviewer #9: Yes

Reviewer #10: Yes

Reviewer #11: Yes

Reviewer #12: Yes

**********

3. Have the authors made all data underlying the findings in their manuscript fully available?

The PLOS Data policy

Reviewer #1: No

Reviewer #2: Yes

Reviewer #3: No

Reviewer #4: No

Reviewer #5: No

Reviewer #6: No

Reviewer #7: Yes

Reviewer #8: Yes

Reviewer #9: No

Reviewer #10: Yes

Reviewer #11: No

Reviewer #12: No

**********

4. Is the manuscript presented in an intelligible fashion and written in standard English?

Reviewer #1: Yes

Reviewer #2: Yes

Reviewer #3: No

Reviewer #4: Yes

Reviewer #5: Yes

Reviewer #6: Yes

Reviewer #7: Yes

Reviewer #8: Yes

Reviewer #9: Yes

Reviewer #10: Yes

Reviewer #11: Yes

Reviewer #12: Yes

**********

Reviewer #1: This is a cross-sectional study with 333 kidney transplant patients in Wuhan, China, to investigate the association between family functioning and resilience with symptom experience. The authors found that resilience plays a mediating role between family functioning and symptom experience of kidney transplant recipients. This is a solid study and analysis, however some inconsistencies and methodological issues warrant further clarification:

1. Sign inconsistencies for complications. It is reported in the text that complications is a strong positive predictor however the coefficient in Table 6 says otherwise. This is a directional error. Please clarify and check coding.

2. Are employment status and monthly income analysed as continuous or categorical in the regression in Table 6? Preferably with dummy variables if it is categorical.

3. A better approach for covariate selection would be based on theory and known confounders rather than p-value. Do clarify or justify method used for model variable selection.

4. Including resilience as a predictor in the hierarchical regression while also treating it as a mediator of family functioning creates a conceptual conflict. Model 2 regression coefficients should be interpreted cautiously. Since resilience is a mediator for family functioning, the authors should mention that model 2 shows the direct effect of family functioning after accounting for resilience, not the full effect. Do discuss the findings from both the hierarchical regression and mediation analysis to tie things together.

Reviewer #2: The topic you have chosen addresses a relevant topic and is supported by an adequate dataset. The study design and analytical approach are appropriate, and the findings are clearly presented. The conclusions are consistent with the results, and data availability requirements have been met.

The manuscript is sound and has the potential to contribute usefully to the field.

Reviewer #3: -I commend the authors' work to investigate how resilience mediates the relationship between family functioning and symptom experience in kidney transplant recipients

-Please find my comments below:

1. page 4 (Ethics Statement field): The form records the ethics statement as "N/A," but the authors report institutional review board approval (Medical Ethics Committee, WDRY2024-K282) and electronic informed consent (manuscript lines 98-100). Please complete the submission form ethics statement to match the manuscript.

2. page 7 (Data Availability Statement): The statement declares that data are available only from the corresponding author on reasonable request and ethics committee approval.

3. Line 3 (author affiliations): The author line contains asterix which is not explained below the author list

4. Line 26 (Abstract, Results): The abstract reports "resilience (β = 0.146, p = .007)" (positive), while the body text (line 211), Table 6 Model 2 (page 19), and Figure 1 (page 21) all report β = −0.146. Please review and correct as appropriate.

5. Lines 61-69: The Theory of Unpleasant Symptoms posits three antecedent classes: physiological, psychological, and situational. This study measures only psychological (resilience) and situational (family functioning) antecedents. Physiological antecedents are not explicitly mentioned. Either acknowledge this as a limitation or add available physiological covariates.

6. Line 81: STROBE adherence is claimed but no checklist is provided. Please include a completed STROBE checklist as supplementary material.

7. Lines 90-96: In addition, the rationale references "approximately 17 demographic, clinical, and psychosocial variables," but the final regression contains only 7 predictors. Please reconcile this discrepancy.

8. Lines 110-111: Transplant duration is collected but does not appear in the regression or mediation models, and several well-established correlates of post-transplant symptom burden are not reported at all: donor type (living vs. deceased), immunosuppression regimen, current eGFR, comorbid diabetes and hypertension. "Complications" is used as a predictor but is not defined. Please specify what counts as a complication, and over what time window, and either include the missing clinical covariates or justify their omission.

9. Line 123: "Items are rated on a 5-point Likert scale", was this rated 1-5, please specify

10.Line 126: Has double period signs.

11. Lines 134-136repeated lines 217-219. Please review and correct

12. Line 126: The sentence "The Family APGAR Index[14] in its Chinese version [15] was employed.." contains a typographical error (double period). Aso, reference 15 (Yu et al., 2024) is a study using the Family APGAR in trauma ICU caregivers, not the primary Chinese psychometric validation. Please cite the original Chinese validation study.

13. Table 2, pages 15-16 (Univariate analysis): some "t" values are quite large for independent-samples t-statistics given the reported means and standard deviations. Please verify each test and report the correct statistic type

14. Lines 178-180: The footnote beneath Table 2 describes skewness and kurtosis thresholds, which is the footnote that belongs to Table 1. Please replace with an appropriate footnote describing the statistical tests used and the reference categories.

15. Line 210: The text states "complications (β = 0.176, p = .001) was the strongest positive predictor of a higher symptom burden." but Table 6 Model 2 displays β = −0.176 for the same predictor. Please review and correct as appropriate.

16. Lines 214-215 (Table 6 footnote): The footnote lists reference groups as "Male, Urban, No Complications." Given the coefficient-sign issue above, please also confirm the reference group.

17. The title is duplicated on lines 239-240 and 241.

18. Lines 295-302: Statements such as a supportive family environment "fosters the development and maintenance of psychological resilience" and that resilience "enables individuals to more effectively appraise and manage illness-related stressors" use causal language that cross-sectional data cannot support. Please soften to associational language and acknowledge plausible reverse-direction pathways (e.g., higher symptom burden eroding resilience and family cohesion).

19. Lines 341-365 about relevance to clinical practice and future research is redundant with lines 306-322. Please consider merging both.

Reviewer #4: Dear Author,

Thank you for such an insightful manuscript.

Your study is relevant and contributes to the growing body of literature on psychosocial determinants of post-transplant outcomes.

Kindly consider going through my recommendations below:

1. Ethics Statement

Please revise the inconsistencies in the ethics statements. The table indicates “N/A,” yet an incomplete ethics statement is included in the methodology. Since this study involves human subjects, “N/A” is not appropriate.

Kindly update the manuscript to include:

The name of the institutional review board or ethics committee that approved the study

The approval number and/or a statement confirming approval

The form of consent obtained (written/oral), or justification if consent was not obtained (e.g., anonymized data)

I have seen some of that information in the methodology but should also be in the table.

2. Data Availability Statement

Please include the name of the institutional ethics committee referred to.

3. Financial Disclosure

The financial disclosure is currently incomplete. Kindly include:

The initials of the authors who received the award

The URL of the funder’s website

A clear statement of the funders’ role in the study (e.g., involvement in study design, data collection and analysis, decision to publish, or manuscript preparation)

4. Bias

Given the use of convenience sampling and a single-center study design, it would be appropriate to acknowledge potential selection/sampling bias, as this affects generalizability and external validity.

5. Grammar and Other Inconsistencies

Please reconcile the coefficient for resilience (β) differing signs between sections:

Abstract: β = 0.146 (positive)

Results: β = –0.146

Line 98: Kindly include the name of the hospital.

Line 126: Remove the double period.

Line 271: Add a full stop after “populations.”

Thank you.

Reviewer #5: Dear Authors,

Thank you for the opportunity to review this manuscript. I commend your efforts and appreciate the focus on resilience, which is a thoughtful and relevant construct in the context of kidney transplant recipients.

While the study offers valuable insights, the cross-sectional design and the potential for subjective bias limit the ability to draw causal inferences. In light of this, I recommend revising the statement “Resilience mediates the relationship between family functioning and symptom experience in kidney transplant recipients” to a more cautious interpretation that reflects an association or potential contribution rather than a definitive mediating effect.

Additionally, although the restriction on data availability for privacy reasons is understandable, it may be beneficial to consider providing an anonymized dataset, if feasible, to align with the journal’s data-sharing requirements and enhance transparency.

Overall, this is an interesting and relevant study, and with these considerations addressed, it could make a meaningful contribution to the literature.

Reviewer #6: This manuscript addresses a clinically relevant topic in kidney transplant recipients and explores the associations among family functioning, resilience, and symptom experience. The study is well organized overall, the sample size is reasonable, and the topic is potentially suitable for publication after substantial revision. However, I have several important concerns that should be addressed before the manuscript can be considered further.

First, there are major inconsistencies in the statistical reporting. In the abstract, resilience is reported as an independent predictor with a positive standardized coefficient, whereas Table 6 reports a negative coefficient for resilience. Similarly, the text states that complications were associated with higher symptom burden, but the regression table shows a negative coefficient for “complications (Yes vs No).” These inconsistencies must be corrected throughout the abstract, results, tables, figure, and discussion so that the direction and interpretation of effects are internally consistent.

Second, the manuscript uses mediation language too strongly for a cross-sectional design. Although an indirect effect can be tested statistically in cross-sectional data, causality and temporal sequence cannot be established. The title, abstract, and discussion should therefore be revised to use more cautious wording, such as findings being consistent with an indirect pathway, rather than asserting a confirmed mediating mechanism.

Third, the statistical approach requires clarification. Table 4 suggests that categorical predictors such as employment status and monthly income may have been entered into regression as ordinal numeric codes. The authors should clearly describe how these variables were modeled and, if necessary, reanalyze them using appropriate dummy coding. In addition, key regression diagnostics are not reported. The manuscript should state whether assumptions such as multicollinearity, linearity, homoscedasticity, and normality of residuals were checked.

Fourth, the manuscript’s data availability statement does not indicate full public availability of the underlying data. Instead, the data are described as available only from the corresponding author upon reasonable request and ethics approval. This should be revised to align with journal policy, or a clearer justification and formal access mechanism should be provided.

Fifth, the ethics reporting appears inconsistent. The manuscript itself reports ethics approval and informed consent, but the submission materials appear to list the ethics statement as “N/A.” This should be corrected to ensure consistent reporting of human-subject ethics approval.

There are also several minor issues. The manuscript would benefit from careful language editing for grammar, punctuation, and repetition. Some parts of the discussion are somewhat speculative and should be toned down or better supported by citations. The figure caption should also clearly indicate whether the coefficients shown are standardized or unstandardized, and the values in the figure should match the corrected analysis.

Overall, the study addresses an important topic, but substantial revision is needed, especially in relation to statistical reporting, analytic clarity, data availability, and the interpretation of mediation in a cross-sectional design.

Reviewer #7: 1. In Line 123, you state the OTSWI is a 40-item instrument on a 5-point Likert scale. This implies a theoretical score range of 40 to 200. In Line 162 and Table 1, the mean score is reported as 46.56 (± 23.86). How can a score of 46.56 be described as a moderate level of symptom burden? Statistically, this is a floor effect. Most participants are reporting almost no symptoms. This undermines the clinical urgency of your integrated interventions.

2. You used a convenience sample from a single tertiary hospital in Wuhan (Line 83). Wuhan's urban healthcare infrastructure differs significantly from rural China. Table 2 shows that "Residence" (Urban vs. Rural) was a significant factor (p=0.005). Does the small rural sub-sample (n=74) provide enough power to justify the claim that your findings apply to the general kidney transplant population?

Reviewer #8: This manuscript describes an important and clinically relevant topic related to psychosocial factors and symptom burden among post-kidney transplant patients. The study is well written with the use of validated assessment tools. However, some minor but important discrepancies and methodological issues need to be addressed before acceptance.

Reviewer #9: Respected authors,

Manuscript has been written in a technically sound manner. I only had two queries: One it is ethically required that despite including in-text citations, additional acknowledgment of developers is required when using questionnaires that are copyright of others. Secondly, the flow of language is so sophisticated that it looks like you have used some ai for editing of your manuscript. It would have been better if you would have declared it in a statement in the methods section.

Reviewer #10: Well-reasoned hypothesis and supported with data. Please confirm the following:

You state that resilience ((beta = 0.146, p = .007)) is an independent predictor of lower symptom burden.

The Problem: If a higher resilience score means more resilience and a higher OTSWI score means more symptoms, the (beta) should be negative (e.g., (-0.146)), just like your correlation ((r = -0.222)). A positive (beta) technically implies that as resilience increases, symptom burden increases. Check your SPSS/SAS output to see if that negative sign was accidentally omitted.

Reviewer #11: I appreciate the opportunity to review this manuscript. The study addresses an important and clinically relevant topic in kidney transplantation, particularly the interplay between family functioning, psychological resilience, and symptom experience. The topic is timely, and the authors have applied appropriate conceptual framing using the Theory of Unpleasant Symptoms, which adds theoretical strength to the work. The manuscript is generally well structured, and the statistical approach is sound, including the use of mediation analysis with bootstrap methods.

My comments are primarily focused on improving clarity, reporting transparency, and methodological detail to enhance reproducibility and interpretability of the findings.

1. Lines 50–51: Please provide a supporting reference for the statement that some patients with apparently good family support still report poor outcomes, as this observation helps justify the proposed mediating mechanism.

2. Lines 112-130: Consider providing representative items or additional scoring details for the instruments in a supplement to improve reproducibility.

3. Please clarify how resilience scores should be interpreted clinically (e.g., whether established cutoffs or normative categories exist for the RS-KTPL-CV).

4. Lines 120-124: Please report the theoretical total score range for the OTSWI and clarify whether higher scores uniformly indicate greater symptom burden across all domains.

5. Lines 147-148: Please specify the criterion used for inclusion of variables from univariate analyses into the multivariable models (e.g., p < 0.05 or another threshold). Additionally, please clarify whether covariate selection was based solely on statistical significance or also informed by theoretical/clinical relevance.

6. Table 1: Consider using more descriptive labels for the OTSWI subdomains (e.g., “sleep disturbance” instead of “sleep”) to improve interpretability for readers unfamiliar with the instrument.

7. References section: Two different references appear to have been assigned the same citation number (19). Please revise the reference numbering for consistency and accuracy.

Reviewer #12: Dear Editor,

Thank you for the opportunity to review the manuscript titled “Resilience mediates the relationship between family functioning and symptom experience in kidney transplant recipients: A cross-sectional study.”

The manuscript examines whether resilience mediates the association between family functioning and symptom experience among adult kidney transplant recipients, using validated Chinese versions of the RS‑KTPL, OTSWI, and Family APGAR, and PROCESS mediation analysis. The research question is timely and clinically meaningful, and the theoretical anchoring in the Theory of Unpleasant Symptoms (TOUS) is appropriate. However, several important methodological and reporting issues require attention before the findings can be interpreted with confidence.

Major comments:

1. The design is cross‑sectional, yet the mediation model is described in strongly causal language (e.g., “resilience mediates,” “effect of family functioning on symptom experience,” “pathway through which family support translates into outcomes”). With simultaneous measurement, the temporal ordering of family functioning → resilience → symptom experience cannot be established.

Although this limitation is mentioned in the discussion, the causal language remains strong. I recommend softening the wording throughout (title, abstract, methods, results, discussion, conclusion) to emphasize “associations,” “indirect statistical pathways,” or “theoretical mediation” rather than causal effects.

It would also be helpful to explicitly state in the methods and discussion that, because the study is cross‑sectional, mediation should be interpreted as a statistical indirect association consistent with the hypothesized TOUS causal ordering framework rather than as evidence of causal mediation.

2. The authors acknowledge the limitation in external validity, but the implications are underdeveloped. There is minimal description of the transplant program context (e.g., typical time post‑transplant, standard immunosuppression protocol at the center, follow‑up schedule, and usual psychosocial support). This makes it difficult for readers to evaluate the generalizability of the findings.

Please add more detail about the clinical setting and care pathways (median/mean time since transplant, standard follow‑up structure, presence and type of formal psychosocial services). In addition, please discuss how single-center convenience sampling and local health‑system characteristics may shape resilience, family functioning, and symptom burden, and be more explicit about the populations to which these results may or may not generalize.

Because participants with psychiatric disorders were excluded from the study sample, the findings may underestimate the relationship between resilience, family functioning, and symptom burden in broader transplant populations. Please discuss how this exclusion criterion may affect external validity and whether psychosocial screening practices at your center differ from those used elsewhere.

3. Sample size was justified using a conventional rule-of-thumb approach, “5–10 participants per independent variable” rule. The text suggests that 17 variables were planned, but it is not entirely clear which variables these are or whether the final models match this plan. Please list all variables included in this calculation and confirm that the final regression and mediation models align with the original analytic plan. If any variables were dropped or added post‑hoc, this should be clearly described and justified.

4. The models control for several socio‑demographic and clinical factors (gender, employment, residence, income, complications), but important potential confounders such as time since transplant, graft function, comorbid mental health diagnoses, and immunosuppressive regimen are not included.

Time since transplant is likely to influence both resilience and symptom experience, and complications are coded as a simple binary variable, which may oversimplify clinical status. Please clarify which clinical variables were collected (e.g., time since transplant, type/number of complications, psychiatric history, graft function indicators) and justify their inclusion or exclusion in the multivariable and mediation models. If possible, sensitivity analyses including key clinical variables (such as time since transplant) would be informative, or at least a statement on whether including them materially changes the estimates for resilience and family functioning.

Regarding the inclusion criterion of “at least 3 months post‑transplant”: Please clarify the rationale for selecting ≥3 months post-transplant as an inclusion criterion and discuss whether symptom burden and resilience may differ during earlier post-transplant phases.

5. The authors rely on Pearson correlations and linear regression, but provide limited information on how assumptions (normality, linearity, homoscedasticity, and multicollinearity) were assessed. Although skewness and kurtosis are reported, they are not clearly linked to the regression assumptions. I recommend briefly describing how regression assumptions were checked (e.g., inspection of residual plots, tests for multicollinearity such as VIFs) and whether any transformations or robust procedures were considered.

6. The indirect effect accounted for 18.4% of the total effect, but the clinical meaning of the effect sizes is not discussed. For instance, what does a 1‑point increase in APGAR or RS‑KTPL correspond to in terms of change in OTSWI score, and is that difference clinically meaningful?

The paper describes a “core contribution” to understanding mechanisms, but the associations between resilience/family functioning and symptom burden are modest, and the model explains only about 14% of the variance in symptoms. The discussion occasionally overstates the model's explanatory contribution, given the modest variance explained. It would be helpful to discuss the practical significance of these effect sizes (e.g., how they compare to known minimal clinically important differences, if available), and to adopt a more modest tone about the explanatory power of the model, emphasizing that resilience and family functioning are part of a broader set of determinants.

Minor comments:

1. Title and abstract: Consider softening the title to reflect association rather than confirmed mediation in a causal sense (e.g., “Resilience and its mediating role in the association between family functioning and symptom experience…”).

In the abstract, please explicitly state that this was a single‑center, convenience sample with a cross‑sectional design, and avoid causal phrasing such as “predictors” or “effect” where not warranted.

2. Introduction: The “paradox” of patients with good family support but poor outcomes is an interesting point; consider citing empirical work in transplantation or similar chronic illness populations that documents this phenomenon.

3. Methods – participants: Clarify the definition and assessment of “major comorbidities or stressful life events” used as exclusion criteria (how were these identified, by whom, and over what time frame?).

Provide more detail on recruitment, including the number of patients approached, the proportion who declined, and the reasons for non‑participation if available.

4. Methods – measures: For each instrument (RS‑KTPL‑CV, OTSWI, APGAR), briefly summarize, where possible, cut‑points indicating low/medium/high levels to help readers interpret the descriptive statistics.

Describe how any reverse‑scored items were handled.

5. Results – descriptive and univariate analyses:

For Table 1, it would be more informative if it presented at first the sample’s socio‑demographic and clinical characteristics (age, sex, income, residence, time since transplant, complications, etc.) and symptom, resilience, and family functioning scores presented in separate table of number 2. Currently, it is difficult to obtain a quick overview of the sample.

You do not need to list skewness and kurtosis for every variable in the table. Instead, state in the methods or results that all key variables showed acceptable skewness and kurtosis, and that the assumptions of approximate normality were met, rather than cluttering the table with these indices.

In Table 2, some note text appears duplicated or misaligned (e.g., RS‑KTPL and APGAR are mentioned in the notes to a table that only shows symptom variables). Please revise table notes so they refer only to what is presented.

6. Results – regression and mediation tables:

In Table 2, it is unclear why “t” and “F” are surrounded by asterisks. Standard practice is to label test statistics simply as t or F, with asterisks used next to p‑values to indicate significance levels. Please clarify and standardize this notation.

In Table 5, several abbreviations (R, R², ΔR², F) are not fully defined. Please spell out what each abbreviation stands for in the table footnote (e.g., R = correlation coefficient; R² = coefficient of determination; ΔR² = change in R²) and explicitly report ΔR² and the F‑change, as these are central to the hierarchical regression.

Table 4 (“Assignment of variables for hierarchical regression”) is essentially methodological. It would improve the flow to move this table (or its contents) to the Methods section or to the supplementary material.

In the text (around line 209), you refer to “the final regression model” but didn’t write “Table 6” in the line of the title of this table.

There appears to be a sign inconsistency between the description in the text and the coefficients for complications in Table 6 (B is negative but is described as a “positive predictor”). Please double-check coding and ensure that the interpretation is clearly explained.

7. Figures and causal structure:

The mediation figure (Figure 1) is helpful; please ensure that all covariates included in the model are listed in the figure legend and consistent with the text.

Because you are testing a mediation model and making assumptions about causal ordering among family functioning, resilience, and symptom experience, including a directed acyclic graph (DAG) or a similar conceptual diagram may improve transparency and strengthen the paper. This could clarify which variables are considered exposures, mediators, outcomes, and confounders.

8. Discussion:

The discussion is rich and engages with the broader literature, but is somewhat long and occasionally speculative, particularly when introducing concepts such as “syndemic” and “secure base”. A more concise discussion, more tightly linked to the actual findings, would improve readability.

Excluding participants with psychiatric disorders may have restricted variability in resilience and symptom experience and may limit applicability to broader transplant populations, in whom anxiety and depression are common.

Overall, this manuscript addresses an important and underexplored aspect of post‑transplant care and uses appropriate, validated instruments and mediation analysis. However, the cross-sectional nature of the data, relatively modest effect sizes, limited control for key clinical confounders, and, at times, overly causal interpretations of mediation warrant substantial revision. With attention to these issues—particularly clarifying the design's causal limitations, improving the description and handling of covariates, and tightening the discussion—the paper could make a useful contribution. I recommend a major revision.

**********

what does this mean?). If published, this will include your full peer review and any attached files.

If you choose “no”, your identity will remain anonymous but your review may still be made public.

**Do you want your identity to be public for this peer review?**  For information about this choice, including consent withdrawal, please see our Privacy Policy

Reviewer #1: No

Reviewer #2: **Yes:**  Dr Surya Bahadur Hamal Thakuri

Reviewer #3: No

Reviewer #4: No

Reviewer #5: **Yes:**  Dr. Oluwafolayemi Doyeni

Reviewer #6: No

Reviewer #7: **Yes:**  sri chakradhar challagali

Reviewer #8: **Yes:**  Dr Zahid Hasan Khan

Reviewer #9: **Yes:**  Saira Akhlaq

Reviewer #10: **Yes:**  Michael Lioudis

Reviewer #11: No

Reviewer #12: No

**********

[NOTE: If reviewer comments were submitted as an attachment file, they will be attached to this email and accessible via the submission site. Please log into your account, locate the manuscript record, and check for the action link "View Attachments". If this link does not appear, there are no attachment files.]

To ensure your figures meet our technical requirements, please review our figure guidelines: https://journals.plos.org/plosone/s/figures

You may also use PLOS’s free figure tool, NAAS, to help you prepare publication quality figures: https://journals.plos.org/plosone/s/figures#loc-tools-for-figure-preparation.

NAAS will assess whether your figures meet our technical requirements by comparing each figure against our figure specifications.

Attachment Submitted filename: Reviewer Report-1.docx

Attachment Submitted filename: Comments.docx

Attachment Submitted filename: Comments on PONE-D-26-00026.docx

# Author comment: Author response to Decision Letter 1

**DOI:** 10.1371/journal.pone.0361968.r002

30 May 2026

We have carefully addressed all comments raised by the Academic Editor and the reviewers. A detailed point-by-point response is provided in the file “Response to Reviewers” (uploaded as a separate document). All changes in the manuscript are highlighted using Track Changes. The Data Availability Statement and Funding Statement have been updated accordingly.

Attachment Submitted filename: Response to Reviewers.docx

# Peer review: Decision Letter 1

**DOI:** 10.1371/journal.pone.0361968.r003

**Authors:** Wisit Kaewput

8 Jul 2026

Dear Dr. TANG,

Thank you for submitting your manuscript to PLOS ONE. After careful consideration, we feel that it has merit but does not fully meet PLOS ONE’s publication criteria as it currently stands. Therefore, we invite you to submit a revised version of the manuscript that addresses the points raised during the review process.

Please submit your revised manuscript by Aug 22 2026 11:59PM. If you will need more time than this to complete your revisions, please reply to this message or contact the journal office at plosone@plos.org. When you're ready to submit your revision, log on to https://www.editorialmanager.com/pone/ and select the 'Submissions Needing Revision' folder to locate your manuscript file.

- A letter that responds to each point raised by the academic editor and reviewer(s). You should upload this letter as a separate file labeled 'Response to Reviewers'.
- A marked-up copy of your manuscript that highlights changes made to the original version. You should upload this as a separate file labeled 'Revised Manuscript with Track Changes'.
- An unmarked version of your revised paper without tracked changes. You should upload this as a separate file labeled 'Manuscript'.

If you would like to make changes to your financial disclosure, please include your updated statement in your cover letter. Guidelines for resubmitting your figure files are available below the reviewer comments at the end of this letter.

If applicable, we recommend that you deposit your laboratory protocols in protocols.io to enhance the reproducibility of your results. Protocols.io assigns your protocol its own identifier (DOI) so that it can be cited independently in the future. For instructions see: https://journals.plos.org/plosone/s/submission-guidelines#loc-laboratory-protocols. Additionally, PLOS ONE offers an option for publishing peer-reviewed Lab Protocol articles, which describe protocols hosted on protocols.io. Read more information on sharing protocols at https://plos.org/protocols?utm_medium=editorial-email&utm_source=authorletters&utm_campaign=protocols.

As the corresponding author, your ORCID iD is verified in the submission system and will appear in the published article. PLOS supports the use of ORCID, and we encourage all coauthors to register for an ORCID iD and use it as well. Please encourage your coauthors to verify their ORCID iD within the submission system before final acceptance, as unverified ORCID iDs will not appear in the published article. *Only*  the individual author can complete the verification step; PLOS staff *cannot*  verify ORCID iDs on behalf of authors.

We look forward to receiving your revised manuscript.

Kind regards,

Wisit Kaewput, MD, FRCP, FRCPSG, FASN, FAcadMEd

Academic Editor

PLOS One

Journal Requirements:

If the reviewer comments include a recommendation to cite specific previously published works, please review and evaluate these publications to determine whether they are relevant and should be cited. There is no requirement to cite these works unless the editor has indicated otherwise.

Please review your reference list to ensure that it is complete and correct. If you have cited papers that have been retracted, please include the rationale for doing so in the manuscript text, or remove these references and replace them with relevant current references. Any changes to the reference list should be mentioned in the rebuttal letter that accompanies your revised manuscript. If you need to cite a retracted article, indicate the article’s retracted status in the References list and also include a citation and full reference for the retraction notice.

[Note: HTML markup is below. Please do not edit.]

Reviewers' comments:

Reviewer's Responses to Questions

**Comments to the Author**

Reviewer #1: All comments have been addressed

Reviewer #3: All comments have been addressed

Reviewer #4: All comments have been addressed

Reviewer #5: All comments have been addressed

Reviewer #9: (No Response)

Reviewer #11: (No Response)

**********

2. Is the manuscript technically sound, and do the data support the conclusions?

Reviewer #1: (No Response)

Reviewer #3: Yes

Reviewer #4: Yes

Reviewer #5: (No Response)

Reviewer #9: Yes

Reviewer #11: Yes

**********

3. Has the statistical analysis been performed appropriately and rigorously?

Reviewer #1: (No Response)

Reviewer #3: Yes

Reviewer #4: Yes

Reviewer #5: (No Response)

Reviewer #9: Yes

Reviewer #11: Yes

**********

4. Have the authors made all data underlying the findings in their manuscript fully available?

The PLOS Data policy

Reviewer #1: (No Response)

Reviewer #3: Yes

Reviewer #4: Yes

Reviewer #5: (No Response)

Reviewer #9: No

Reviewer #11: No

**********

5. Is the manuscript presented in an intelligible fashion and written in standard English?

Reviewer #1: (No Response)

Reviewer #3: Yes

Reviewer #4: Yes

Reviewer #5: (No Response)

Reviewer #9: Yes

Reviewer #11: Yes

**********

Reviewer #1: (No Response)

Reviewer #3: I commend the authors for the efforts in addressing reviewer comments. Thank you for addressing all comments.

Reviewer #4: Dear Author(s),

Thank you for carefully revising the manuscript. You have addressed the majority of the comments, and the revisions have strengthened both the clarity and the overall impact of the study.

I just have only a few minor comments:

• Please ensure that the Ethics Statement is fully harmonized across the manuscript and the submission information, particularly between the ethics table and the "Ethical Approval" section where there is "N/A" in the table yet a description under the ethical approval.

• I recommend one final round of proofreading to correct minor formatting and grammatical inconsistencies, particularly around citations, spacing, and punctuation.

For example:

○ Line 311: There appears to be a missing full stop after "populations"

○ Line 369: "such dual-path approach"; I would think there is a missing article, maybe "such a dual-path approach" ?

○ Line 370: "potential mitigating symptoms"; I would suggest "potentially"

I wish you all the best with your publication.

Reviewer #5: The limitations of causal inferences have been addressed.

There are no additional comments at the moment.

Thank you

Reviewer #9: Respected authors,

You have addressed my previous comments to some extent. Even though there are not copyrights apparently for the questionnaires you have used. However, in order to give full credit, ideally you should give a link to from where you downloaded the questionnaire and under acknowledgment write sentences thanking the original developers of the study. Even though you used modified version, you still need to acknowledge the original developer. Secondly, you need to attach in the appendix the permission letter that you obtained from one of the developers. Thirdly, your references are not in accordance with the Vancouver style. They do not need PMCID numbers at the end.

Reviewer #11: 1) Lines 141-142: In the Methods, the description of the scoring contains a grammatical inconsistency. I would suggest revising for clarity.

2) I appreciate the authors' response; however, I respectfully disagree and would ask them to reconsider. My concern is twofold.

First, the labels currently used in Table 1 do not in fact correspond to the original OTSWI subdomain labels. The validated instrument and its referenced version use designations such as 'Ache of lower limb,' 'Cognitive functioning,' 'Basic activities in daily life,' and 'Economy,' whereas Table 1 uses 'Pain,' 'Cognition,' 'Activity,' and 'Economic issues.' The authors have therefore already departed from the original labels, so the stated rationale of preserving consistency with the source instrument does not hold as written.

Second, several of the abbreviated labels are ambiguous to readers. For example, 'Activity' does not convey that this subdomain concerns limitations in basic activities of daily living (e.g., showering, dressing, shopping due to physical condition); ‘Sleep’ does not convey that this subdomain concerns sleeping problems as used in the original OTSWI instrument.

**********

what does this mean?). If published, this will include your full peer review and any attached files.

If you choose “no”, your identity will remain anonymous but your review may still be made public.

**Do you want your identity to be public for this peer review?**  For information about this choice, including consent withdrawal, please see our Privacy Policy

Reviewer #1: No

Reviewer #3: No

Reviewer #4: No

Reviewer #5: No

Reviewer #9: **Yes:**  Saira Akhlaq

Reviewer #11: No

**********

[NOTE: If reviewer comments were submitted as an attachment file, they will be attached to this email and accessible via the submission site. Please log into your account, locate the manuscript record, and check for the action link "View Attachments". If this link does not appear, there are no attachment files.]

To ensure your figures meet our technical requirements, please review our figure guidelines: https://journals.plos.org/plosone/s/figures

You may also use PLOS’s free figure tool, NAAS, to help you prepare publication quality figures: https://journals.plos.org/plosone/s/figures#loc-tools-for-figure-preparation.

NAAS will assess whether your figures meet our technical requirements by comparing each figure against our figure specifications.

# Author comment: Author response to Decision Letter 2

**DOI:** 10.1371/journal.pone.0361968.r004

18 Jul 2026

Response to Reviewers

Manuscript ID: PONE-D-26-00026R1

Title: Resilience and its mediating role in the association between family functioning and symptom experience in kidney transplant recipients: A cross-sectional study

Dear Dr. Kaewput and Reviewers,

Thank you for your further evaluation of our revised manuscript. We are grateful for the remaining constructive comments, which have helped us further improve the paper. Below we provide point‑by‑point responses to all new comments raised in this round. All changes are highlighted in the revised manuscript using Track Changes.

Response to Reviewer #4

Comment #1 (Ethics Statement harmonization): Please ensure that the Ethics Statement is fully harmonized across the manuscript and the submission information, particularly between the ethics table and the "Ethical Approval" section where there is "N/A" in the table yet a description under the ethical approval.

Our response: We confirm that the “N/A” entry in the submission system refers only to the absence of a specified expiration date in the ethics approval document issued by the Medical Ethics Committee of Renmin Hospital of Wuhan University. This is consistent with the committee’s standard practice for non‑interventional studies (cross‑sectional questionnaire surveys), where approval is granted without a fixed expiry date, provided the study is conducted in accordance with the approved protocol. The ethics approval itself (Approval No. WDRY2024‑K282) and the informed consent procedures are fully described in the manuscript (“Ethical approval” section).

Comment #2 (Language proofreading): I recommend one final round of proofreading to correct minor formatting and grammatical inconsistencies, particularly around citations, spacing, and punctuation. For example: Line 311 missing full stop; Line 369 missing article; Line 370 word form.

Our response: We thank the reviewer for the careful reading. We have corrected all the identified issues: (1) added a full stop after “populations” in the discussion of gender differences; (2) changed “such dual-path approach” to “such a dual-path approach”; (3) changed “potential mitigating” to “potentially mitigating.” We have also performed a final proofread of the entire manuscript to address other minor formatting and grammatical inconsistencies.

Response to Reviewer #9

Comment #1 (Questionnaire acknowledgment): In order to give full credit, ideally you should give a link to from where you downloaded the questionnaire and under acknowledgment write sentences thanking the original developers of the study. Even though you used modified version, you still need to acknowledge the original developer.

Our response: We agree with the reviewer and have added formal acknowledgment of the original developers of all three instruments (RS-KTPL, OTSWI, and Family APGAR) in the Acknowledgments section at the end of the manuscript. We also thank the translators of the Chinese version of the OTSWI in the same section.

Comment #2 (Permission letter): You need to attach in the appendix the permission letter that you obtained from one of the developers.

Our response: We do not have a formal written permission letter to attach. We confirm the following: (1) The Family APGAR is a public‑domain instrument and does not require permission; (2) The RS‑KTPL‑CV was cross‑culturally adapted and validated by our own research team [13], and we acknowledge the original developers in the Acknowledgments; (3) The Chinese version of the OTSWI was obtained from the translators for academic research purposes and was used in accordance with its intended use, with appropriate citation provided [14]. As there was no formal written permission letter, we are unable to include one in the supplementary materials. We have, however, added clear acknowledgments of the original developers in the manuscript to give full credit.

Comment #3 (Reference format – Vancouver style, remove PMCID): Your references are not in accordance with the Vancouver style. They do not need PMCID numbers at the end.

Our response: We have revised all references according to PLOS ONE’s Vancouver style requirements. All PubMed PMID and PMCID numbers have been removed, and only essential citation information (authors, title, journal, year, volume, pages, DOI) is retained.

Response to Reviewer #11

Comment #1 (Grammar in Methods scoring description, Lines 141-142): The description of the scoring contains a grammatical inconsistency.

Our response: We have revised the scoring description in the Methods section from “Higher total scores indicating a greater symptom burden” to “Higher total scores indicate a greater symptom burden” to correct the grammatical inconsistency.

Comment #2 (OTSWI subdomain labels in Table 1): The labels currently used in Table 1 do not in fact correspond to the original OTSWI subdomain labels... several of the abbreviated labels are ambiguous to readers.

Our response: We thank the reviewer for this careful observation. We acknowledge that the previously used labels were descriptive summaries rather than the original factor labels. To address this, we have revised the Table 1 subdomain labels to align with the seven‑factor structure of the Chinese version of the OTSWI as reported by Shi et al. The revised labels are: Sleeping problems, Joint and muscle pain, Fatigue, Cognitive functioning, Basic ADL, Mood, and Economy.

Additional Note

During this revision, the affiliation of the first and corresponding author (Dr. Yan Tang) has been updated to reflect her current institution: Hemodialysis Center, The Sixth Hospital of Wuhan, Affiliated Hospital of Jianghan University, Wuhan, China. All other authors’ affiliations remain unchanged. This change has been made in both the manuscript and the submission system.We hope that the revised manuscript is now acceptable for publication in PLOS ONE. Thank you again for your time and consideration.

Sincerely,

Yan Tang, on behalf of all authors

Hemodialysis Center, The Sixth Hospital of Wuhan, Affiliated Hospital of Jianghan University, Wuhan, China

E‑mail: 1348580254@qq.com

Attachment Submitted filename: Response to Reviewers-7.9.docx

# Peer review: Decision Letter 2

**DOI:** 10.1371/journal.pone.0361968.r005

**Authors:** Wisit Kaewput

10 Sep 2026

Dear Dr. TANG,

Thank you for submitting your manuscript to PLOS One. After careful consideration, we feel that it has merit but does not fully meet PLOS One’s publication criteria as it currently stands. Therefore, we invite you to submit a revised version of the manuscript that addresses the points raised during the review process.

Please submit your revised manuscript by Oct 25 2026 11:59PM. If you will need more time than this to complete your revisions, please reply to this message or contact the journal office at plosone@plos.org. When you're ready to submit your revision, log on to https://www.editorialmanager.com/pone/ and select the 'Submissions Needing Revision' folder to locate your manuscript file.

- A letter that responds to each point raised by the academic editor and reviewer(s). You should upload this letter as a separate file labeled 'Response to Reviewers'.
- A marked-up copy of your manuscript that highlights changes made to the original version. You should upload this as a separate file labeled 'Revised Manuscript with Track Changes'.
- An unmarked version of your revised paper without tracked changes. You should upload this as a separate file labeled 'Manuscript'.

If you would like to make changes to your financial disclosure, please include your updated statement in your cover letter. Guidelines for resubmitting your figure files are available below the reviewer comments at the end of this letter.

If applicable, we recommend that you deposit your laboratory protocols in protocols.io to enhance the reproducibility of your results. Protocols.io assigns your protocol its own identifier (DOI) so that it can be cited independently in the future. For instructions see: https://journals.plos.org/plosone/s/submission-guidelines#loc-laboratory-protocols. Additionally, PLOS One offers an option for publishing peer-reviewed Lab Protocol articles, which describe protocols hosted on protocols.io. Read more information on sharing protocols at https://plos.org/protocols?utm_medium=editorial-email&utm_source=authorletters&utm_campaign=protocols.

As the corresponding author, your ORCID iD is verified in the submission system and will appear in the published article. PLOS supports the use of ORCID, and we encourage all coauthors to register for an ORCID iD and use it as well. Please encourage your coauthors to verify their ORCID iD within the submission system before final acceptance, as unverified ORCID iDs will not appear in the published article. *Only*  the individual author can complete the verification step; PLOS staff *cannot*  verify ORCID iDs on behalf of authors.

We look forward to receiving your revised manuscript.

Kind regards,

Wisit Kaewput, MD, FRCP, FRCPSG, FASN, FAcadMEd

Academic Editor

PLOS One

Journal Requirements:

If the reviewer comments include a recommendation to cite specific previously published works, please review and evaluate these publications to determine whether they are relevant and should be cited. There is no requirement to cite these works unless the editor has indicated otherwise.

Please review your reference list to ensure that it is complete and correct. If you have cited papers that have been retracted, please include the rationale for doing so in the manuscript text, or remove these references and replace them with relevant current references. Any changes to the reference list should be mentioned in the rebuttal letter that accompanies your revised manuscript. If you need to cite a retracted article, indicate the article’s retracted status in the References list and also include a citation and full reference for the retraction notice.

Additional Editor Comments:

The manuscript has improved substantially and most previous concerns have been adequately addressed. However, a few issues should be clarified before acceptance.

1. Please explicitly confirm whether there is any participant overlap between the present cohort of 333 kidney transplant recipients and the cohort reported in Tang et al. (2025). If overlap exists, please report its extent and clarify the relationship between the two datasets.

2. Please recheck the statistical values reported in Tables 2 and 5 against the original output. Some values appear internally inconsistent, including several t statistics in Table 2 and the negative t value reported for a positive regression coefficient for complications in Table 5.

3. Please clarify how employment status and monthly income were coded in the regression analyses. As these are multi-category variables, particularly employment status, appropriate indicator/dummy coding should be used. If they were entered as single numerical variables, the analyses should be revised accordingly.

If these issues are satisfactorily addressed and the main findings remain unchanged, the manuscript would be suitable for acceptance.

[Note: HTML markup is below. Please do not edit.]

Reviewers' comments:

Reviewer's Responses to Questions

**Comments to the Author**

Reviewer #9: All comments have been addressed

Reviewer #11: (No Response)

**********

2. Is the manuscript technically sound, and do the data support the conclusions?

Reviewer #9: Yes

Reviewer #11: Yes

**********

3. Has the statistical analysis been performed appropriately and rigorously?

Reviewer #9: Yes

Reviewer #11: Yes

**********

4. Have the authors made all data underlying the findings in their manuscript fully available?

The PLOS Data policy

Reviewer #9: No

Reviewer #11: No

**********

5. Is the manuscript presented in an intelligible fashion and written in standard English?

Reviewer #9: Yes

Reviewer #11: (No Response)

**********

Reviewer #9: Good amendments overall. However, before publishing please confirm the copyright rules and regulations as you do not have the permission letter.

Reviewer #11: I thank the authors for correcting the verb form. Several related points remain, and I would ask for clarification before this is finalized.

1) First, the scoring metric described in the Methods differs from that of the Chinese version cited as the source [14], in which items are scored 0–4, the item means reported in that validation study range from 0.097 to 1.602, which is not attainable under 1-to-5 scoring, giving a total range of 0–160 rather than 40–200. If the rescoring was intentional, it should be stated explicitly and its consequences for comparability with the source instrument acknowledged.

Second, the values in Table 1 do not appear compatible with 1-to-5 scoring in any case. Under that scheme the minimum possible score for a subscale equals its number of items, yet Basic ADL (three items in the cited version) has a reported mean of 1.05, below the minimum attainable score of 3. I would ask the authors to confirm which metric was used in the analysis and to reconcile the Methods, Table 1, and the derived statement in the Discussion ("46.56/200"). The characterization of symptom burden also needs attention: the Results describe it as "moderate" (line 192) while the Limitations describe it as "relatively low" (line 379), and under 1-to-5 scoring a total of 46.56 would lie only 6.56 points above the theoretical minimum of 40.

2) "Joint and muscle pain" is a descriptive summary rather than the label used in the source instrument ("Ache of lower limb"). Please correct the name of this label.

**********

what does this mean?). If published, this will include your full peer review and any attached files.

If you choose “no”, your identity will remain anonymous but your review may still be made public.

**Do you want your identity to be public for this peer review?**  For information about this choice, including consent withdrawal, please see our Privacy Policy

Reviewer #9: **Yes:**  Saira Akhlaq

Reviewer #11: No

**********

[NOTE: If reviewer comments were submitted as an attachment file, they will be attached to this email and accessible via the submission site. Please log into your account, locate the manuscript record, and check for the action link "View Attachments". If this link does not appear, there are no attachment files.]

To ensure your figures meet our technical requirements, please review our figure guidelines: https://journals.plos.org/plosone/s/figures

You may also use PLOS’s free figure tool, NAAS, to help you prepare publication quality figures: https://journals.plos.org/plosone/s/figures#loc-tools-for-figure-preparation.

NAAS will assess whether your figures meet our technical requirements by comparing each figure against our figure specifications.

# Author comment: Author response to Decision Letter 3

**DOI:** 10.1371/journal.pone.0361968.r006

25 Sep 2026

Thank you for the further evaluation of our manuscript. We have now addressed all remaining comments and uploaded the revised files via the Editorial Manager system. We confirmed that the two datasets were collected at different time periods; although partial overlap cannot be excluded, the studies address different questions and are not duplicate publications. We rechecked and corrected all statistical values in Tables 2 and 5, and re-ran the hierarchical regression using dummy coding for employment status and monthly income, as requested. The updated results are presented in the revised Tables 4 and 5, and the main findings remain unchanged: resilience (β = −0.152, p = .005) and family functioning (β = −0.124, p = .022) remained significant negative predictors, while complications remained a significant positive predictor (β = 0.169, p = .001). We also corrected the OTSWI scoring description (0–4 scale, total 0–160), revised the subdomain label to “Ache of lower limb,” confirmed the copyright status of all instruments, and added appropriate acknowledgments. A detailed point-by-point response is provided in the “Response to Reviewers” file. Thank you again for your time and consideration.

Attachment Submitted filename: Response_to_Reviewers_auresp_3.docx

# Editor report: Decision Letter 3

**DOI:** 10.1371/journal.pone.0361968.r007

**Authors:** Wisit Kaewput

6 Oct 2026

Dear Dr. TANG,

Thank you for submitting your manuscript to PLOS One. After careful consideration, we feel that it has merit but does not fully meet PLOS One’s publication criteria as it currently stands. Therefore, we invite you to submit a revised version of the manuscript that addresses the points raised during the review process.

Please submit your revised manuscript by Nov 20 2026 11:59PM. If you will need more time than this to complete your revisions, please reply to this message or contact the journal office at plosone@plos.org. When you're ready to submit your revision, log on to https://www.editorialmanager.com/pone/ and select the 'Submissions Needing Revision' folder to locate your manuscript file.

- A letter that responds to each point raised by the academic editor and reviewer(s). You should upload this letter as a separate file labeled 'Response to Reviewers'.
- A marked-up copy of your manuscript that highlights changes made to the original version. You should upload this as a separate file labeled 'Revised Manuscript with Track Changes'.
- An unmarked version of your revised paper without tracked changes. You should upload this as a separate file labeled 'Manuscript'.

If you would like to make changes to your financial disclosure, please include your updated statement in your cover letter. Guidelines for resubmitting your figure files are available below the reviewer comments at the end of this letter.

If applicable, we recommend that you deposit your laboratory protocols in protocols.io to enhance the reproducibility of your results. Protocols.io assigns your protocol its own identifier (DOI) so that it can be cited independently in the future. For instructions see: https://journals.plos.org/plosone/s/submission-guidelines#loc-laboratory-protocols. Additionally, PLOS One offers an option for publishing peer-reviewed Lab Protocol articles, which describe protocols hosted on protocols.io. Read more information on sharing protocols at https://plos.org/protocols?utm_medium=editorial-email&utm_source=authorletters&utm_campaign=protocols.

As the corresponding author, your ORCID iD is verified in the submission system and will appear in the published article. PLOS supports the use of ORCID, and we encourage all coauthors to register for an ORCID iD and use it as well. Please encourage your coauthors to verify their ORCID iD within the submission system before final acceptance, as unverified ORCID iDs will not appear in the published article. *Only*  the individual author can complete the verification step; PLOS staff *cannot*  verify ORCID iDs on behalf of authors.

We look forward to receiving your revised manuscript.

Kind regards,

Wisit Kaewput, MD, FRCP, FRCPSG, FASN, FAcadMEd

Academic Editor

PLOS One

Journal Requirements:

If the reviewer comments include a recommendation to cite specific previously published works, please review and evaluate these publications to determine whether they are relevant and should be cited. There is no requirement to cite these works unless the editor has indicated otherwise.

Please review your reference list to ensure that it is complete and correct. If you have cited papers that have been retracted, please include the rationale for doing so in the manuscript text, or remove these references and replace them with relevant current references. Any changes to the reference list should be mentioned in the rebuttal letter that accompanies your revised manuscript. If you need to cite a retracted article, indicate the article’s retracted status in the References list and also include a citation and full reference for the retraction notice.

Additional Editor Comments:

The manuscript has been substantially improved and the major methodological concerns have been adequately addressed. However, a few internal inconsistencies remain that should be resolved before acceptance.

Please undertake one final careful check of the revised manuscript against the original statistical output. In particular:

1. The regression coefficients and p values for resilience and family functioning reported in the response letter do not appear to match those reported in the revised manuscript. Please verify the final values against the original SPSS output and ensure that the Abstract, Results, Tables, and response letter are fully consistent.

2. The text states that residence was included among the demographic and clinical variables entered into Model 1, whereas residence does not appear in the Table 4 footnote or Table 5. Please clarify whether residence was included in the final regression model and revise the Methods, Results, and tables accordingly.

3. In Table 5, “Employment: Other vs Other” appears to be a labeling error and should presumably read “Employed vs Other.” Please verify and correct this.

4. Please ensure that the description of the relationship between the present cohort and the previous Tang et al. (2025) study accurately reflects your response that partial participant overlap cannot be excluded.

These are primarily consistency and reporting issues and do not require additional external review. Once they have been satisfactorily addressed, the manuscript should be suitable for acceptance.

[Note: HTML markup is below. Please do not edit.]

[NOTE: If reviewer comments were submitted as an attachment file, they will be attached to this email and accessible via the submission site. Please log into your account, locate the manuscript record, and check for the action link "View Attachments". If this link does not appear, there are no attachment files.]

To ensure your figures meet our technical requirements, please review our figure guidelines: https://journals.plos.org/plosone/s/figures

You may also use PLOS’s free figure tool, NAAS, to help you prepare publication quality figures: https://journals.plos.org/plosone/s/figures#loc-tools-for-figure-preparation.

NAAS will assess whether your figures meet our technical requirements by comparing each figure against our figure specifications.

# Author comment: Author response to Decision Letter 4

**DOI:** 10.1371/journal.pone.0361968.r008

7 Oct 2026

We have addressed all four comments from the Academic Editor. Below is a point-by-point response.

Comment 1: The regression coefficients and p values for resilience and family functioning reported in the response letter do not appear to match those reported in the revised manuscript. Please verify the final values against the original SPSS output and ensure that the Abstract, Results, Tables, and response letter are fully consistent.

Our response: We have rechecked all values against the original SPSS output. In the Results text, the values had not been updated and still read: complications (β = 0.176, p = .001), resilience (β = −0.146, p = .007), family functioning (β = −0.123, p = .023). These have now been corrected to: complications (β = 0.169, p = .001), resilience (β = −0.152, p = .005), family functioning (β = −0.124, p = .022). The Abstract and Table 5 already contained the correct values. The Abstract, Results, Tables, and this response letter are now fully consistent.

Comment 2: The text states that residence was included among the demographic and clinical variables entered into Model 1, whereas residence does not appear in the Table 4 footnote or Table 5. Please clarify whether residence was included in the final regression model and revise the Methods, Results, and tables accordingly.

Our response: We confirm that residence was included in both Model 1 and Model 2. We have added the residence row to Table 5 (Model 1: B = 6.186, SE = 3.240, β = 0.108, t = 1.909, p = .057, 95% CI [−0.19, 12.56]; Model 2: B = 5.950, SE = 3.178, β = 0.104, t = 1.872, p = .062, 95% CI [−0.30, 12.20]). The Table 4 footnote now reads: “Model 1 predictors: gender, employment status (dummy variables), residence, monthly income (dummy variables), and complications.”

Comment 3: In Table 5, “Employment: Other vs Other” appears to be a labeling error and should presumably read “Employed vs Other.” Please verify and correct this.

Our response: Corrected. The row previously labeled “Employment: Other vs Other” has been changed to “Employment: Employed vs Other.” The reference category remains “Other” for employment status.

Comment 4: Please ensure that the description of the relationship between the present cohort and the previous Tang et al. (2025) study accurately reflects your response that partial participant overlap cannot be excluded.

Our response: We have added a statement in the Limitations section: “The present cohort partially overlaps with the sample reported in our prior methodological study (Tang et al., 2025), which focused on the validation of the RS-KTPL instrument. Because the two studies recruited from the same outpatient clinic at different time periods, partial participant overlap cannot be excluded. However, the two studies address different research questions and report different outcomes.”

Attachment Submitted filename: point-by-point response-10.8..docx

# Editor report: Decision Letter 4

**DOI:** 10.1371/journal.pone.0361968.r009

**Authors:** Wisit Kaewput

20 Oct 2026

Resilience and its mediating role in the association between family functioning and symptom experience in kidney transplant recipients: A cross-sectional study

PONE-D-26-00026R4

Dear Dr. TANG,

We’re pleased to inform you that your manuscript has been judged scientifically suitable for publication and will be formally accepted for publication once it meets all outstanding technical requirements.

Within one week, you’ll receive an e-mail detailing the required amendments. When these have been addressed, you’ll receive a formal acceptance letter and your manuscript will be scheduled for publication.

An invoice will be generated when your article is formally accepted. Please note, if your institution has a publishing partnership with PLOS and your article meets the relevant criteria, all or part of your publication costs will be covered. Please make sure your user information is up-to-date by logging into Editorial Manager at Editorial Manager® and clicking the ‘Update My Information' link at the top of the page. For questions related to billing, please contact billing support.

If your institution or institutions have a press office, please notify them about your upcoming paper to help maximize its impact. If they’ll be preparing press materials, please inform our press team as soon as possible -- no later than 48 hours after receiving the formal acceptance. Your manuscript will remain under strict press embargo until 2 pm Eastern Time on the date of publication. For more information, please contact onepress@plos.org.

Kind regards,

Wisit Kaewput, MD, FRCP, FRCPSG, FASN, FAcadMEd

Academic Editor

PLOS One

Additional Editor Comments (optional):

The authors have satisfactorily addressed the four outstanding editorial comments in this fourth revision. The regression coefficients and corresponding p-values have been reconciled, the residence variable has been incorporated into the regression tables, the employment-category labeling error has been corrected, and the potential participant overlap with the previous publication has been appropriately acknowledged.

The revised manuscript demonstrates substantial improvement in reporting consistency and methodological transparency. The limitations of the cross-sectional design and the interpretation of statistical mediation are also appropriately acknowledged.

I have no further substantive methodological concerns requiring another round of external peer review. I recommend acceptance, subject only to final verification that the corrected regression coefficients are present in Table 5 and that the description of potential cohort overlap is consistent across the submission files.

Reviewers' comments:

The authors have satisfactorily addressed the four outstanding editorial comments in this fourth revision. The regression coefficients and corresponding p-values have been reconciled, the residence variable has been incorporated into the regression tables, the employment-category labeling error has been corrected, and the potential participant overlap with the previous publication has been appropriately acknowledged.

The revised manuscript demonstrates substantial improvement in reporting consistency and methodological transparency. The limitations of the cross-sectional design and the interpretation of statistical mediation are also appropriately acknowledged.

I have no further substantive methodological concerns requiring another round of external peer review. I recommend acceptance, subject only to final verification that the corrected regression coefficients are present in Table 5 and that the description of potential cohort overlap is consistent across the submission files.

# Editor report: Acceptance letter

**DOI:** 10.1371/journal.pone.0361968.r010

**Authors:** Wisit Kaewput

PONE-D-26-00026R4

PLOS One

Dear Dr. TANG,

I'm pleased to inform you that your manuscript has been deemed suitable for publication in PLOS One. Congratulations! Your manuscript is now being handed over to our production team.

At this stage, our production department will prepare your paper for publication. This includes ensuring the following:

* All references, tables, and figures are properly cited

* All relevant supporting information is included in the manuscript submission,

* There are no issues that prevent the paper from being properly typeset

You will receive further instructions from the production team, including instructions on how to review your proof when it is ready. Please keep in mind that we are working through a large volume of accepted articles, so please give us a few days to review your paper and let you know the next and final steps.

Lastly, if your institution or institutions have a press office, please let them know about your upcoming paper now to help maximize its impact. If they'll be preparing press materials, please inform our press team within the next 48 hours. Your manuscript will remain under strict press embargo until 2 pm Eastern Time on the date of publication. For more information, please contact onepress@plos.org.

You will receive an invoice from PLOS for your publication fee after your manuscript has reached the completed accept phase. If you receive an email requesting payment before acceptance or for any other service, this may be a phishing scheme. Learn how to identify phishing emails and protect your accounts at https://explore.plos.org/phishing.

If we can help with anything else, please email us at customercare@plos.org.

Thank you for submitting your work to PLOS One and supporting open access.

Kind regards,

PLOS One Editorial Office Staff

on behalf of

Dr. Wisit Kaewput

Academic Editor

PLOS One
